# Protocol for high-resolution 3D spatial transcriptomics using Open-ST

**DOI:** 10.1016/j.xpro.2024.103521

**Published:** 2024-12-19

**Authors:** Marie Schott, Daniel León-Periñán, Elena Splendiani, Elisabetta Ferretti, Giuseppe Macino, Nikos Karaiskos, Nikolaus Rajewsky

**Affiliations:** 1 Laboratory for Systems Biology of Regulatory Elements, Berlin Institute for Medical Systems Biology (BIMSB), Max-Delbrück-Centrum for Molecular Medicine in the Helmholtz Association (MDC), Hannoversche Str. 28, 10115 Berlin, Germany; 2 Department of Experimental Medicine, Sapienza University, Rome, Italy; 3 Department of Cellular Biotechnologies and Hematology, La Sapienza University of Rome, 00161 Rome, Italy; 4 Charité - Universitätsmedizin, Charitéplatz 1, 10117 Berlin, Germany; 5 German Center for Cardiovascular Research (DZHK), Site Berlin, Berlin, Germany; 6 NeuroCure Cluster of Excellence, Berlin, Germany; 7 German Cancer Consortium (DKTK), Heidelberg, Germany; 8 National Center for Tumor Diseases (NCT), Site Berlin, Berlin, Germany

**Keywords:** bioinformatics, cancer, RNA-seq, microscopy, systems biology

## Abstract

Spatial transcriptomics (ST) is fundamental for understanding molecular mechanisms in health and disease. Here, we present a protocol for efficient and high-resolution ST in 2D/3D with Open-ST. We describe all steps for repurposing Illumina flow cells into spatially barcoded capture areas and preparing ST libraries from stained cryosections. We detail the computational workflow for generating 2D/3D molecular maps (“virtual tissue blocks”), aligned with histological data, unlocking molecular pathways in space. Open-ST is applicable to any tissue, including clinical samples.

For complete details on the use and execution of this protocol, please refer to Schott et al.^1^

## Highlights

• Convert NGS flow cells into high-resolution spatial transcriptomics capture areas
• Generate spatial transcriptomics libraries from stained and imaged cryosections
• Modular pipeline for preprocessing, automated alignment, and segmentation
• Create 3D virtual tissue blocks from serial tissue sections using open-source tools

## Abstract (editor highlights)

Publisher’s note: Undertaking any experimental protocol requires adherence to local institutional guidelines for laboratory safety and ethics.

## Graphical abstract

**Figure:**
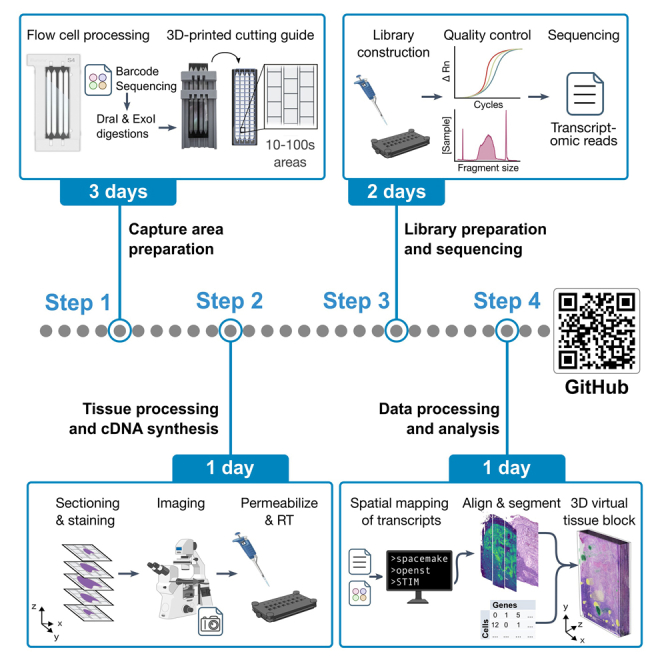

## Before you begin

Spatial transcriptomics (ST) has emerged as a crucial tool for understanding the complex organization of tissues in health and disease, revealing how the spatial arrangement of cells and their gene expression patterns contribute to organ function and pathology. Open-ST addresses this need by providing a cost-effective method for whole-transcriptome capture at subcellular resolution, by converting patterned Illumina flow cells into ST capture areas with ∼0.6 μm barcoded spots. This enables the study of diverse tissue types from tumor microenvironments to structured organs, in 2D and 3D. Importantly, Open-ST is a fully open-source and adaptable resource for researchers across various fields of biology and medicine.

This protocol covers the implementation of Open-ST. The workflow covers (1) generating capture areas, (2) preparing libraries, and (3) analyzing data in both 2D and 3D contexts, including the reconstruction of 3D "virtual tissue blocks" from serial sections.

We demonstrate the protocol using human metastatic lymph node tissue from a head and neck squamous cell carcinoma patient, showcasing Open-ST’s capabilities from 2D sections to a 3D reconstruction from serial sections. The method has been applied to diverse tissues, including mouse embryonic head, adult mouse hippocampus, and various human clinical samples,^1^ and can be applied to any species.

Before you begin, we recommend carefully selecting your tissue sample: tissue must be freshly frozen and embedded in OCT. The RNA quality, expressed as the RNA integrity number (RIN), should ideally be > 7. Furthermore, choose and embed your tissue to maximize the tissue coverage of your Open-ST capture area, in order to increase library input.

We advise a pilot experiment to compare different permeabilization conditions using a qPCR assay. We suggest comparing different pepsin incubation times (for example, 0, 15, 30, 45, and 60 min) at 37°C. For permeabilization optimization, follow the library preparation steps until qPCR for cycle number assessment. Earlier amplification corresponds to a higher concentration of starting material, i.e., more efficient mRNA capture. If conditions amplify together, choose the shorter time or lower pepsin concentration for permeabilization of your sample.

Moreover, to support the implementation of our method, we provide a list of tissues that were effectively analyzed using our protocol, along with the corresponding permeabilization conditions for each tissue type (Table 1 “Permeabilization conditions for tissue types tested’’).

**Table 1:** Permeabilization conditions for tissue types tested

Species	Tissue type	Timing (min)	Additional protocol adaptation
Mouse	Brain (E13)	30	–
	Brain (Adult)	30	–
Human	Metastatic lymph node	45	1.4 U/μL pepsin
	Healthy lymph node	45	1.4 U/μL pepsin
	Head and neck squamous cell carcinoma tumor	45	1.4 U/μL pepsin

We maintain an up-to-date, open-source version of our methodology on our online resources (https://rajewsky-lab.github.io/openst). We refine and expand our protocol based on new developments and user feedback. We encourage researchers to engage with us through communication channels available on our website to improve and adapt Open-ST across diverse research applications.

### Institutional permissions

The present protocol uses human tissue samples. Before working with patient material, ensure that adequate permissions from relevant institutions have been acquired and that patients have given informed consent.

This study was performed according to the ethical principles for medical research of the Declaration of Helsinki and approval was approved by the Ethics Committee of the Charité University Medical Department in Berlin (EA4/082/22).

### Tissue freezing and embedding

**Timing: 20 min**

1. Freeze and embed the tissue as soon as possible upon collection.

***Note:*** Ideally, embed the tissue to allow maximum coverage of the capture areas when sectioning. When the tissue is small (<1 mm^2^), such as some organoids or punch biopsies, embed multiple samples in close proximity. If the tissue is > 0.5 mm wide or high, consider cutting it into multiple pieces and embedding these separately.

2. Keep the tissue on ice until freezing/embedding.
3. Under a chemical hood, fill a metal beaker with 50–100 mL of isopentane.
4. Place the beaker into a box filled with liquid nitrogen or, if not available, dry ice (minimally at the same level as the isopentane).
5. Let the isopentane cool down for 10–15 min. Close the box with a lid to limit evaporation.

**CRITICAL:** Ensure that the isopentane has cooled to the temperature of the dry ice or liquid nitrogen. When using dry ice, this can be tested by placing a piece of dry ice into the isopentane; if the isopentane boils, it is not yet cold enough.

6. Whilst the isopentane is cooling, choose the appropriate tissue cryomold based on tissue dimensions and label it.
7. Add a drop of OCT into the cryomold avoiding bubbles.
8. Place the tissue into the cryomold and add OCT until the cryomold is filled and the tissue is covered. If bubbles occur close to the tissue, remove using a pipette tip.
9. Using forceps place the cryomold into the cold isopentane, without fully submerging it. Try to keep the top of the cryomold level with the surface of the isopentane.
10. Wait 1–2 min until the OCT is completely frozen (solid and white).
11. Store the sample in an air-tight container, such as a zip-lock bag, at −80°C.

***Note:*** You can embed a tissue sample that was already frozen starting from step 3. However, due to partial rethawing of the tissue when placed into OCT, we recommend simultaneous freezing and embedding where possible.

**Pause point:** The embedded tissue can be stored at −80°C long-term.

### RNA quality control

**Timing: 2 h +**

We recommend assessing the RNA integrity of your tissue prior to performing Open-ST. As Open-ST is based on poly-dT capture, degradation of RNA will affect the capture efficiency. For best results we recommend using tissues with a RIN >7.

We acknowledge that RNA integrity is dependent on tissue type and the logistics of processing (time from collection, freezing and embedding simultaneously or consecutively) and that human tissue can be challenging to obtain. The metastatic lymph node referred to in this protocol had a RIN of 6.3 and still yielded high-quality Open-ST data.

**CRITICAL:** Work in an RNase-free environment and use filter tips throughout. Clean all tools used during cryosectioning (brushes, tweezers) with RNaseAway or other solutions for nuclease removal.

***Note:*** Excess OCT negatively affects RNA extraction. If OCT is abundant in your sections, reduce it by trimming it off with a razor. You may also centrifuge the sample after adding your first extraction buffer. The excess OCT will pellet; continue the RNA extraction with the supernatant.

12. Using a cryostat, cut 3–4 30 μm sections of the OCT-embedded tissue.
13. Transfer the sections into a pre-cooled tube (temperature of cryostat or dry ice), using pre-cooled forceps or a brush.
14. Keep the sections frozen until starting your extraction protocol.
15. Extract the RNA using your preferred method (column-based kit or manual phase separation).
16. Measure the concentration using a spectrophotometer.
17. Assess the RNA integrity (RIN) using automated gel electrophoresis (e.g., Bioanalyzer or Tapestation).

### 3D printing of the flow cell cutting tool

**Timing: printer-specific, ∼5 h**

We have designed cutting guides that facilitate the breaking of the Illumina NovaSeq 6000 SP and S4 flow cells into regular capture areas. These cutting guides, and an optional breaking aid, can be 3D-printed using the .stl files provided (Items S1, S2, and S3).

18. Find a 3D printing service near you.
19. Print your tool(s) using the .stl file(s) provided.
20. File down any protruding filaments if required.
21. Before use, clean the cutting guide with RNase-away or a similar solution for RNase-removal and 80% Ethanol.

***Note:*** Various 3D printers and slicing softwares can be used. We printed the cutting guide with 1.75 mm polylactic acid (PLA) filament on the Pro 3 Dual Extruder 3D printer from Raise 3D. The .stl file was prepared for printing using IdeaMaker v.4.3.2 as the 3D slicing software.

### Barcode sequencing for Open-ST capture area generation

**Timing: ∼14 h**

***Note:*** We recommend quantifying the HDMI-32-DraI library using the KAPA library quantification kit (Roche).

22. Sequence your HDMI32-DraI library on an Illumina NovaSeq 6000 S4 flow cell (35 cycles), at a loading concentration of 200 pM using the custom Read1-DraI primer and the custom sequencing recipe (Item S4).
23. After sequencing, retrieve the flow cell.
24. Obtain basecall files from the sequencing run (processed in step 86).

**CRITICAL:** The custom sequencing recipe was used in a sequencing run with the following versions and may require adjustment for use with different consumable or software versions: RTA v.3.4.4, Flow Cell Consumable v.1, Sbs Consumable v.3, NovaSeq control Software v.1.7.5. Consult the Illumina technical support if an update is required. You can also consider physically exchanging the bleach solution for water to avoid the use of custom recipes.^2^

***Note:*** You can also select a different Illumina NovaSeq 6000 patterned flow cell (S4, S2, S1, SP). Different flow cells will require different custom sequencing recipes and cutting guides, and differ in the dimensions of the imaged area. Here, we describe the use of the S4 flow cell. In Items S2, S5 and S6, the use of the Illumina NovaSeq 6000 SP flow cell is detailed.

**Pause point:** You can store the flow cell at +4°C before processing. We have stored it for 1 month.

## Key resources table

**Table:** 

REAGENT or RESOURCE	SOURCE	IDENTIFIER
**Biological samples**		

Human head and neck squamous cell carcinoma metastatic lymph node tissue	Schott et al.^1^	N/A

**Chemicals, peptides, and recombinant proteins**		

RNase AWAY	Thermo Fisher Scientific	Cat#7003
Dra I enzyme	NEB	Cat#R0129
Alkaline phosphatase calf intestinal (CIAP) enzyme	Promega	Cat#M1821
Exonuclease I enzyme	NEB	Cat#M0293
NaOH solution, for molecular biology, 10 M in H_2_O	Sigma	Cat#72068
UltraPure 1 M Tris-HCl pH 7.5	Invitrogen	Cat#15567027
Tissue-Tek OCT	Sakura	Cat#4583
Tissue-Tek Cryomold	Sakura	Cat#4565 or 4566 or 4557
2-Methylbutane (Isopentane), ReagentPlus, ≥99%	Sigma	Cat#M32631
MX35 Ultra microtome blades	Epredia	Cat#3053835
Methanol (> 99.8%)	Th. Geyer	Cat#1437
2-propanol (>99.9%)	Th. Geyer	Cat#1197
Acetic acid, glacial, ≥99%	VWR Chemicals	Cat#20102.292
Haematoxylin, Mayer’s	Agilent Dako	Cat#S3309
Bluing buffer	Agilent Dako	Cat#CS702
Eosin Y, aqueous	Sigma	Cat#HT110216
Pepsin from porcine gastric mucosa	Sigma	Cat#P7000
20× SSC	Sigma	Cat#S6639-1L
Hydrochloric acid (HCl), 10 N	AppliChem	Cat#187051
Syringe filter, 0.22 μm, sterile, 25 mm	VWR	EU Cat#514-1263
Luer-lock syringe, 50 mL	Becton Dickinson	GTIN30382903096542
BSA molecular biology grade (conc. 20 mg/mL)	NEB	Cat#B9000S
dNTP SET 100 mM 4× 1 mL	Life Technologies	Cat#R0182
SuperScript IV reverse transcriptase	Life Technologies	Cat#18090010
RiboLock RNase inhibitor	Thermo Scientific	Cat#EO0381
Tris-HCl buffer, pH 8.0, 1 M	Life Technologies	Cat#AM9855G
Sodium chloride NaCl (5 M), RNase-free	Invitrogen	Cat#AM9760G
Roti-Stock 20% SDS ready-to-use, sterile filtered	Roth	Cat#1057.1
UltraPure 0.5 M EDTA, pH 8.0 4× 100 mL	Life Technologies	Cat#15575020
Proteinase K (800 mU/μL)	NEB	Cat#P8107S
DNA Polymerase Large Fragment exo- Klenow Fragment (3′-5′ exo-)	NEB	Cat#M0212
Ampure XP beads	Beckman Coulter	Cat#A63881
Kapa HiFi Hotstart Readymix, KK2612	Roche	Cat#7958960001
1.5% Agarose cassettes, dye-free, int. Stds BluePippin, 250 bp–1.5 kb, Marker R2 or 1.5% Agarose, PippinHT, 300–1500 bp	Biozym	Cat#342BDF1510 or 343HTC1510
Qubit dsDNA HS Assay Kit	Invitrogen	Cat#Q32854
High sensitivity DNA kit	Agilent	Cat#5067-4626
HS RNA TapeStation	Agilent	Cat#5067–5579/ - 5580/ −5581
Blue S'Green qPCR mix	Biozym	Cat#331416
KAPA Library Quantification Primer + Mastermix (Illumina/ LC480)	Roche	Cat#7960573001
KAPA Library Quantification DNA Standards (Illumina)	Roche	Cat#7960387001
Ethanol denatured 96%	Serva Electrophoresis	Cat#11096.02

**Critical commercial assays**		

NovaSeq 6000 S4 reagent kit v.1.5 (35 cycles)	Illumina	Cat#20044417

**Deposited data**		

Open-ST	GEO	GSE251926

**Oligonucleotides**		

HDMI32-DraI: CAAGCAGAAGACGGCATACGAGATTC TTTCCCTACACGACGCTCTTCCGATCTNNVNBVNNVN NVNNVNNVNNVNNVNNVNNNNNTCTTGTGACTACA GCACCCTCGACTCTCGCTTTTTTTTTTTTTTTTTTTTTTT TTTTTTTAAAGACTTTCACCAGTCCATGATGTGTAGAT CTCGGTGGTCGCCGTATCATT (synthesis as Ultramer DNA oligos by IDT or EXTREmer by Eurofins recommended, standard desalting, use standard mixed bases for the barcode synthesis)	Cho et al.^3^	N/A
Randomer: TCAGACGTGTGCTCTTCCGATCTNNNNNNNNN (standard desalted)	Cho et al.^3^	N/A
Read1-DraI: ATCATGGACTGGTGAAAGTCTTT AAAAAAAAAAAAAAAAAAAAAAAAAAAAAA GCGAGAGTCGAGGGTGCTGTAGTCACAAGA (synthesis as Ultramer DNA oligos by IDT or EXTREmer by Eurofins recommended, standard desalting)	Cho et al.^3^	N/A
P5 Fwd: AATGATACGGCGACCACCGAGATCTACACTC TTTCCCTACACGACGCTCT∗T∗C (∗denotes phosphorothioated DNA bases, order HPLC purified)	Cho et al.^3^	N/A
P7 Rev indexing: CAAGCAGAAGACGGCATACGAGAT [8-mer index sequence] GTGACTGGAGTTCAGACG TGTGCTCTTCC∗G∗A (∗denotes phosphorothioated DNA bases, order HPLC purified)	8-mer i7 index sequences from the Nextera XT Index Kit v2 Cho et al.,^3^; Illumina Inc^4^	N/A

**Software and algorithms**		

Illumina Sequencing Analysis Viewer (SAV)	V.3.0.0	https://support.illumina.com/sequencing/sequencing_software/sequencing_analysis_viewer_sav/downloads.html
IdeaMaker v.4.3.2	Raise3D	https://www.raise3d.com/download/
Fiji v.1.53t	Schindelin et al.^5^	https://imagej.net/software/fiji/
BZ-X Viewer 1.3.1.1	Keyence	N/A
BZ-X Analyzer 1.4.1.1	Keyence	N/A
Paraview v.5.11.0	Ahrens et al.^6^	https://www.paraview.org/
Cellpose v.2.2	Pachitariu et al.^7^	https://github.com/MouseLand/cellpose
STAR v.2.7.10b	Dobin et al.^8^	https://github.com/alexdobin/STAR
Bowtie2 v.2.5.1	Langmead et al.^9^	https://github.com/BenLangmead/bowtie2
Drop-seq tools v.2.5.1	^10^	https://github.com/broadinstitute/Drop-seq
spacemake v.0.7.9	Sztanka-Toth et al.^11^	https://github.com/rajewsky-lab/spacemake
scanpy v.1.9.3	Wolf et al.^12^	https://github.com/scverse/scanpy
scikit-image v.0.19.3	van der Walt et al.^13^	https://github.com/scikit-image/scikit-image
STIM v.0.3.0	Preibisch et al.^14^	https://github.com/PreibischLab/STIM
samtools v.1.17	Li et al.^15^	https://github.com/samtools/samtools
scipy v.1.10.0	Virtanen et al.^16^	https://github.com/scipy/scipy
Kornia v.0.7.0	Riba et al.^17^	https://github.com/kornia/kornia
openst v.0.2.3	Schott et al.^1^	https://github.com/rajewsky-lab/openst
napari 0.4.19.post1	Chiu et al.^18^	https://napari.org/stable/
StepOne Software v.2.3	Applied Biosystems	N/A
2100 Expert version B.02.10.SI764	Agilent	N/A

**Other**		

16-Well ProChamber microarray system	Grace Bio-Labs	Cat#645508
Kimtech wipes	Kimberly-Clark	Cat#12538187
STERICAN no. 20 single use needles, 0.40 × 20 mm	Braun	Cat#4657705
DNA LoBind tubes 1,5 mL	VWR	Cat#525-0130
Tungsten carbide tip scriber	BioTrend	Cat#MD9-29
Glass cutter, 138°	Toyo	Cat#TC17B
SMD Tweezers set	Wiha	Cat#32349, EAN 4010995323493
Cutting guide 3D-print *.stl* file for NovaSeq S4 flow cell (Illumina)	This publication, updated from^1^	https://rajewsky-lab.github.io/openst
Raise 3D printer with dual extruder	Raise 3D	Raise 3D Pro3
Mastercycler X50s	Eppendorf	Cat#6311000010
StepOnePlus Real-Time PCR System	Applied Biosystems	Cat#43-766-00
LightCycler 480	Roche	Cat#05015278001
HybEZ II Oven (220 VAC) and HybEZ Humidity Control Tray with lid	ACDBio	Cat#. 321720 and Cat# 310012
Keyence BZ-X710	Keyence	-
CryoStar NX70 cryostat with Cold Disinfection and Vacutome	Epredia	Cat#957070
2100 Bioanalyzer	Agilent	Cat#G2939BA
BluePippin	Sage Science	Cat#BLU0001
Nanodrop-1000 spectrophotometer	Thermo Fisher Scientific	-
Qubit 4 Fluorometer	Thermo Fisher Scientific	Cat#Q33238
NovaSeq 6000	Illumina	-

## Materials and equipment

• Work in an RNase-free environment for capture area generation and library preparation until after reverse transcription. Clean tools and surfaces with RNase AWAY.

***Alternatives:*** Other commercial solutions for RNase decontamination can be used.

• Preparation of 0.45 M Tris-acetic acid buffer at pH 6.0: Dissolve 5.5 g Tris base in 50 mL nuclease-free water. Adjust the pH to 6.0 with 100% acetic acid and bring the volume to 100 mL with nuclease-free water. Filter the solution through a 0.22 μm filter and store at room temperature.
• Preparation of 2× SSC at pH 2.5: Add 10 mL 20× SSC to 50 mL nuclease-free water. Adjust the pH to 2.5 with 10 N hydrochloric acid and bring the volume to 100 mL with nuclease-free water. Filter the solution through a 0.22 μm filter and store at room temperature.
• Preparation of 0.1 M NaOH solution (800 mL): Dilute 8 mL 10 M NaOH in 792 mL nuclease-free water. Mix and store at room temperature. Always prepare on the same day as usage.
• Preparation of 0.1 M Tris-HCl (pH 7.5) solution (800 mL): Dilute 80 mL 1 M Tris-HCl (pH 7.5) in 720 mL nuclease-free water. Mix and store at room temperature.
• Preparation of 80% Ethanol (10 mL): Add 2 mL of nuclease-free water in 8 mL of 99% EtOH. Always prepare on the same day as usage.
• Preparation of Tris-EDTA (TE) buffer (Final concentration 10 mM Tris-HC1, 1 mM EDTA, pH 8.0) (10 mL): Dilute 100 μL 1M Tris-HCl Buffer (pH 8.0) and 20 μL 0.5 M EDTA (pH 8) in 9,880 μL nuclease-free water. Filter the solution through a 0.22 μm filter and store at room temperature.

**CRITICAL:** Prepare the DraI mix, reverse transcription mix, exonuclease I mixes, 2nd strand synthesis mix, qPCR mix and library amplification mix on the same day as usage and store at 4°C. Prepare the buffered Eosin and tissue removal mix on the same day as usage and store at room temperature.

**Table:** DraI mix

Reagent	Final concentration	Volume per Lane (μL)
DraI	2 U/μL	15
10× CutSmart buffer	1×	15
Nuclease-free water	–	120
Total	–	150

Store at 4°C and prepare on the day of usage.

**Table:** Exonuclease I mix for flow cell processing

Reagent	Final concentration	Volume per Lane (μL)
10× ExoI buffer	1 U/μL	15
Exonuclease I (20 U/μL)	1×	7.5
Calf intestinal phosphatase (1 U/μL)	0.05 U/μL	7.5
Nuclease-free water	–	120
Total	–	150

Store at 4°C and prepare on the day of usage.

**Table:** Buffered eosin

Reagent	Volume (μL)
Eosin Y	500
0.45 M Tris-Acetate buffer pH 6.0	500
**Total**	**1000**

Store at room temperature and prepare on the day of usage.

**Table:** Reverse transcription (RT) buffer

Reagent	Final concentration	Volume (μL)
5× SuperScript IV RT Reaction Buffer	1×	20
Ribolock (40 U/μL)	1 U/μL	2.5
Nuclease-free water	N/A	77.5
Total	–	100

Store at 4°C and prepare on the day of usage.

**Table:** Reverse transcription mix

Reagent	Final concentration	Volume (μL)
5× SuperScript IV RT Reaction Buffer	1×	20
0.1 M DTT	5 mM	5
BSA (20 mg/mL)	0.187 mg/uL	0.93
10 mM dNTP mix	1 mM	10
Superscript IV (200 U/μL)	6.67 U/μL	3.33
Ribolock (40 U/μL)	1 U/μL	2.5
Nuclease-free water	N/A	58.24
Total	–	100

Store at 4°C and prepare on the day of usage.

**Table:** Exonuclease I mix for library preparation

Reagent	Final concentration	Volume (μL)
10× Exo I buffer	1×	10
Exonuclease I (20 U/μL)	1 U/μL	5
Nuclease-free water	N/A	85
Total	–	100

Store at 4°C and prepare on the day of usage.

**Table:** Tissue removal mix

Reagent	Final concentration	Volume (μL)
1 M Tris-HCl pH 8.0	100 mM	10
5 M NaCl	200 mM	4
20% SDS	2%	10
0.5 M EDTA	5 mM	1
Proteinase K (800 mU/μL)	16 mU/μL	2
Nuclease-free water	N/A	73
Total	–	100

Store at room temperature and prepare on the day of usage.

**Table:** Second strand synthesis mix

Reagent	Final concentration	Volume (μL)
10× NEBuffer-2	1×	10
100 μM randomer	10 μM	10
10 mM dNTPs	1 mM	10
Klenow exo (−) Fragment (5 U/μL)	0.5 U/μL	10
Nuclease-free water	N/A	60
Total	–	100

Store at 4°C and prepare on the day of usage.

**Table:** qPCR master mix

Reagent	Final concentration	Volume (μL)
2×Blue S'Green qPCR mix + ROX	1×	10
10 μM p5_fwd primer	1 μM	2
10 μM p7_rev_indexing primer	1 μM	2
Nuclease-free water	N/A	3.5
Total	–	17.5

Store at 4°C and prepare on the day of usage.

***Note:*** The addition of ROX passive dye depends on your qPCR instrument. For the StepOnePlus Real-Time PCR System (Applied Biosystems) we add 35 μL of 50 μM ROX additive to 1 mL 2× Blue S'Green qPCR mix.

**Table:** Library amplification mix

Reagent	Final concentration	Volume (μL)
2× KAPA HiFi Hotstart Readymix	1×	100
100 μM p5_fwd primer	1 μM	2
100 μM p7_rev_indexing primer	1 μM	2
Purified 2^nd^ strand product	N/A	80
Nuclease-free water	N/A	16
Total	–	200

Store at 4°C and prepare on the day of usage.

• Raise 3D printer with dual extruder (Raise 3)

***Alternatives:*** Any other 3D printer can be used.

• Nanodrop 1000 (Thermo Fisher Scientific).

***Alternatives:*** Any other spectrophotometer equipped for RNA quantification can be used.

• Keyence BZ-X710 (Keyence) microscope

***Alternatives:*** Any other bright-field microscope with motorized acquisition.

• 16-Well ProChamber Microarray System (Grace Bio-Labs)

***Alternatives:*** ProPlate 16 Well Slide Module (#204862, Grace Bio-Labs), 12 Well Chamber, removable (Ibidi, Cat.No:81201); further well dimensions are available from the respective manufacturers.

• PCR cycler (Mastercycler X50s, Eppendorf).

***Alternatives:*** Any other thermal cycler can be used.

• StepOnePlus Real-Time PCR System (Applied Biosystems).

***Alternatives:*** LightCycler 480 (Roche) or any other qPCR machine can be used.

• 2100 Bioanalyzer (Agilent)

***Alternatives:*** Tapestation4200 (2991BA, Agilent), 5300 Fragment Analyzer (M5311AA, Agilent) or any other automated electrophoresis instrument can be used.

• Bluepippin (Sage science)

***Alternatives:*** PippinHT (HTP0001, Sage science) or manual extraction from agarose gel (DNA Gel Extraction Kit, Norgen Bioteck #SKU 13100 or similar kit can be used).

• NovaSeq 6000 sequencer (Illumina)

***Alternatives:*** This protocol has been developed using the NovaSeq6000 for capture area generation. For Open-ST library sequencing the NextSeq 2000, NovaSeq X Plus or any Illumina high-throughput Next-Generation sequencers can be used.

## Step-by-step method details

### Preparation of Open-ST capture areas

**Timing: 2 days**

These steps describe the processing of the NovaSeq 6000 S4 barcoded flow cell from “before you begin” Step 23 to generate ready-to-use capture areas for Open-ST library preparation. Approximately 360 capture areas at 3 × 4 mm can be generated from one NovaSeq 6000 S4 flow cell, however, it is possible to make pieces of different dimensions up to a maximum size of 7 × 89 mm (the uninterrupted sequenced area of the flow cell).

***Note:*** As an alternative we detail the use of the Illumina NovaSeq 6000 SP flow cell, which requires less initial financial investment. Please refer to Items S2, S5, and S6 for the 3D-printed cutting guide, the SP sequencing recipe and details on the flow cell processing.

**CRITICAL:** Work in an RNase-free environment. Clean surfaces and tools with RNase AWAY or other equivalent cleaning solutions and use filter tips.

***Note:*** Add or remove solutions by placing a P1000 pipette into the inlet or outlets at the start or end of each lane. Pipette slowly; you will see the liquid flow through the lane and exit from the opposite hole.

1. Wash the flow cell by adding and removing 150 μL nuclease-free water per lane, three times.
2. Prepare the DraI reaction mix freshly.

***Note:*** Prepare mixes in excess. ∼150 μL per lane is required for the S4 flow cell.

**Figure 1:**
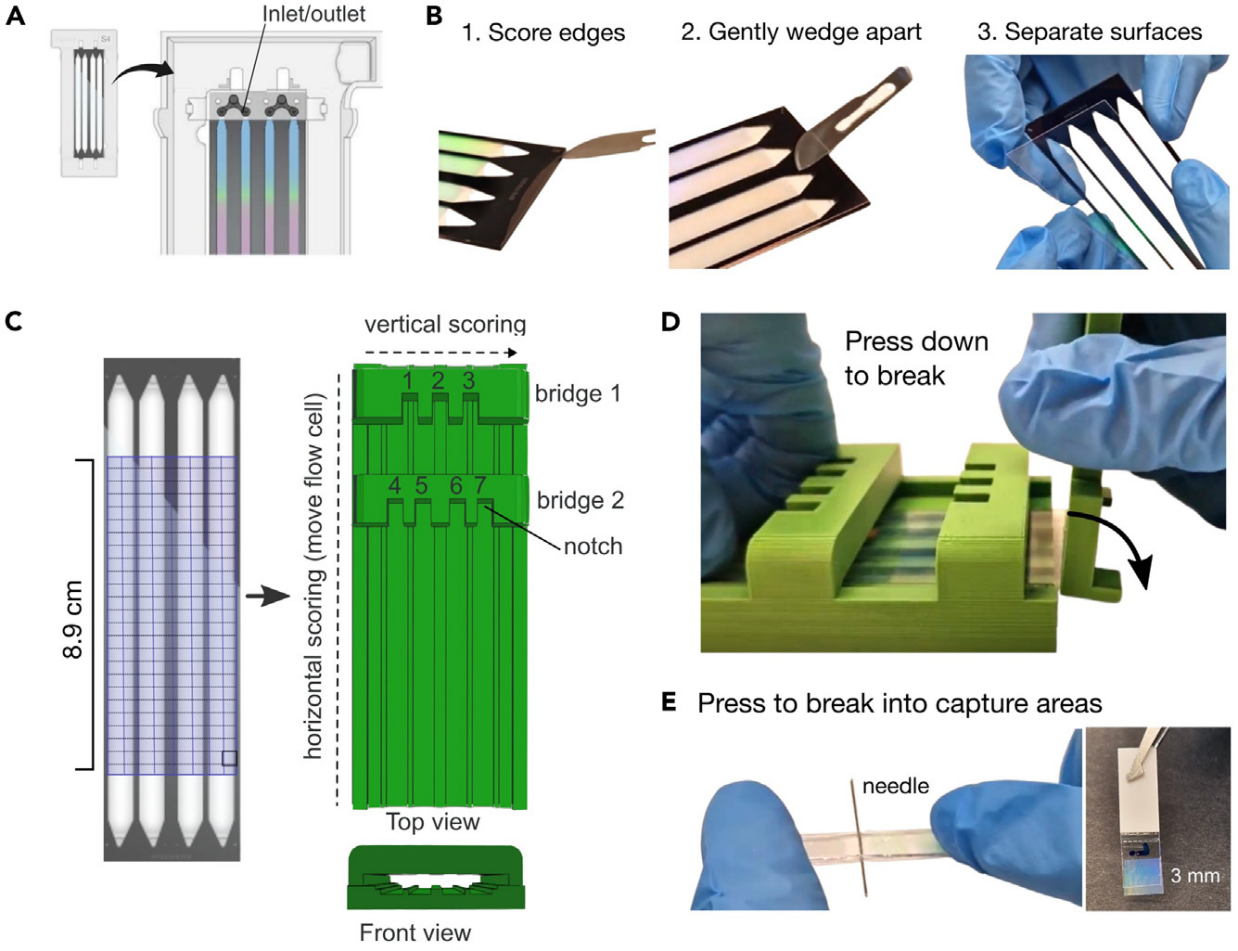
Flow cell opening and capture area generation (A) Illumina NovaSeq 6000 S4 flow cell in its plastic support, with inlet/outlet of lanes indicated. Image courtesy of Illumina. (B) Opening of a NovaSeq 6000 flow cell (here, S4). (C) Design of the 3D-printable cutting guide. Left: Dicing scheme on flow cell. Right: Top and front view of cutting guide. (D) Process of breaking of flow cell using the cutting guide and hand-held breaking aid. (E) Breaking of the scored flow cell slide over a needle (left). Photo of a resulting capture area (right).
13. Perform denaturation with 0.1 M sodium hydroxide to obtain single stranded capture oligos.
  a. Wash the two flow cell surfaces in a beaker of 800 mL freshly prepared 0.1 N NaOH, incubating for 5 min.
  b. Wash the surfaces in a beaker of 800 mL 0.1 M Tris-HCl (pH 7.5).
  c. Wash the surfaces in a beaker of nuclease-free water.
14. Let the flow cell surfaces air dry before proceeding with the scoring and breaking of the flow cell surfaces into smaller capture areas.
15. Score and break the flow cell surfaces into smaller capture areas for use, using the 3D-printable tool as a cutting guide for use with a hand-held glass cutter (Item S1). Troubleshooting 2.
  ***Optional:*** You can practice the flow cell opening and cutting on used (waste) flow cells.
  ***Note:*** The cutting of the top and bottom flow cell layers is described separately, as their handling differs due to their difference in thickness.
  **CRITICAL:** For both flow cell layers, remove the first and last 1.5 cm of the lanes, as these areas are not imaged by the sequencer and thus do not contain registered barcodes.
  ***Note:*** The scores in the glass will be the path of least resistance along which the flow cell can be broken.
  a. Score and break the bottom (thick) flow cell layer into smaller capture areas. (Video S1)
    i. Slide the bottom (thick) flow cell layer with the barcodes (active side) facing down into the 3D-printed cutting guide.
    ii. Place the hand-held glass cutter into a notch of the 3D-printed cutting guide.
    iii. Position the flow cell layer so that the glass cutter is positioned at one end of the flow cell.
    iv. Applying even pressure with the glass cutter, slide the flow cell layer along the cutting guide creating a single score along the length of the flow cell.
    v. Repeat steps i-iv six times, each time placing the glass cutter into a different notch of the cutting tool.
      ***Note:*** If you do not wish to divide each lane horizontally, you can skip the scoring with notches 4–7 (Figure 1C). This will result in 7 mm long (6.3 mm imaged) capture areas.
    vi. Measure and mark 1.5 cm from both ends of the lane (iridescent area). These will be cut off and removed.
    vii. Sliding the hand-held glass cutter along the edge of bridge 1 of the cutting tool creates a vertical score across the flow cell.
      ***Optional:*** Mark the top of the flow cell with a pen to help with the identification of the top/bottom surface. We suggest drawing a small “L.” for “lower” on the glass areas between the lanes for orientation (Figure 1E).
    viii. Align the vertical score with the cutting guide edge and using something straight-edged (like our hand-held breaking aid) apply even pressure onto the overhanging flow cell. It will break along the vertical score.
    ix. Repeat vii-viii along the length of the flow cell. First break off 1.5 cm of the lane on one end for disposing, then continue with vertical scoring to create slices of 4 mm width.
    x. Align a score of the resulting 4 mm slice (active side down) over a needle. Apply gentle pressure using your fingers to break the piece along the score using the needle as a pivot point.
      **CRITICAL:** Do not slide the flow cell slices on your working area. Move them using tweezers, to avoid scratches.
    xi. Repeat for all scores until you obtain eight 3 × 4 mm capture areas per 4 mm slice.
  b. Score and break the top (thin) flow cell layer into smaller capture areas.
    ***Note:*** The top (thin) flow cell layer is easy to break. Apply light pressure throughout.
    i. Perform steps 14 a (i-iv) on the top (thin) flow cell layer, for notches 1–3, only (scoring horizontally between lanes).
    ii. Place the top flow cell layer barcode face down onto a dust-free wipe.
    iii. Using a tungsten scribe and a ruler, score the flow cell as desired.
    iv. Use the cutting guide and hand-held breaking aid to break off vertical slices along the length of the flow cell.
    v. Perform steps 14a (x-xi) to obtain small capture areas.
16. Stick the capture area pieces (with the capture oligos facing up) onto a piece of cut-up plate-sealing tape to facilitate the handling. (Video S2) Troubleshooting 3.
17. Wash the capture areas with nuclease-free water before use.

***Note:*** It is possible to distinguish the active side from the bottom by its iridescent appearance (Figure 1E). If you are unable to detect the active side, please refer to troubleshooting section, problem 3.

3. Add 150 μL DraI mix into each lane. Troubleshooting 1.
4. Remove the flow cell from its plastic encasing and close the inlet and outlet holes with plate-sealing tape to prevent evaporation.
5. Incubate at 37°C overnight (15–18 h).
6. Remove the tape and reassemble the flow cell into its plastic encasing.
7. Wash the flow cell lanes by slowly flowing through 150 μL of 80% ethanol per lane.
8. Wash the flow cell lanes by slowly flowing through 150 μL of nuclease-free water three times per lane.
9. Freshly prepare Exonuclease I mix.
10. Add the Exonuclease I mix and incubate for 45 min at 37°C. Troubleshooting 1.
11. Wash flow cell lanes by flowing through 150 μL of nuclease-free water three times.
12. Open the flow cell by separating the two glass surfaces (Video S1, Figure 1B).
  ***Note:*** The flow cell is composed of three layers: a top (thin) flow cell glass, a black plastic spacer, and a bottom (thick) flow cell glass.
  **CRITICAL:** Remove the liquid from the flow cell lanes as best as possible. Otherwise, the liquid will seep between the flow cell layers, making their separation more challenging.
  a. Remove the flow cell from its plastic encasing.
  b. Carefully score along the sides of the flow cell using a scalpel, keeping the blade in one plane with the flow cell.
    ***Note:*** You can see the detachment of the black plastic layer from the flow cell glass (Figure 1B). Try to score between the black plastic spacer and the same glass layer. This allows you to visually follow the detachment on all sides, without having to turn over the flow cell horizontally.
    **CRITICAL:** Do not slide the scalpel past the black layer into the flow cell lanes. This is not required and can lead to breaking of the top (thin) flow cell layer or scratching of the flow cell surface.
  c. Once all sides detach, carefully separate the two glass layers of the flow cell.
  d. Peel off the black plastic spacer from the glass layer that it remained attached to.
    **Pause point:** You can store the opened (dry) flow cell at +4°C before further processing. We have stored it for up to 1 month.

**Pause point:** We have stored the capture areas dry at −20°C for one year without observing a decrease in library quality.

Video S1. Breaking the flow cell into capture areas, related to Methods “Preparation of Open-ST capture areas,” steps 12–15

Video S2. Capture area handling, related to “Preparation of Open-ST capture areas,” step 15

### Tissue sectioning and fixation

**Timing: 1–3 h**

This section describes the cryosectioning of the tissue sample and the transferal of sections to the capture area.

***Optional:*** Before sectioning onto the capture areas you may wish to assess the histology and define the tissue region-of-interest if planning to select only part of the tissue area. For this perform sectioning, fixation, staining and imaging (steps 18–37) using microscope slides.

***Note:*** We recommend working with up to 8 sections at once (from sectioning, step 22, until reverse transcription, step 47), to limit waiting times before fixation and during imaging and possible RNA degradation.

**CRITICAL:** Pre-cool methanol to −20°C before starting with tissue sectioning.

18. Place the OCT-embedded fresh frozen tissue in a cryostat for 20 min to allow it to warm to the selected cutting temperature (adjusted according to tissue).

***Note:*** For the metastatic lymph node we used a blade temperature of −30°C and a cryostat chamber temperature of −16°C.

19. Prepare 2 mL tubes with 1 mL 100% methanol for tissue fixation. Pre-cool to −20°C.
20. Place the capture areas at room temperature.
21. Section the tissue at 10 μm thickness.
22. Place the capture area onto the tissue section on the cutting stage. The tissue will melt onto the capture area (Video S3).

**CRITICAL:** The transfer of the tissue must be done precisely and quickly. As soon as the tissue melts onto the capture area, remove the capture area from the cryostat stage to avoid the re-freezing of the tissue onto the stage.

***Note:*** Trim the excess OCT surrounding the tissue to prevent folding of OCT over/under the tissue. Alternatively, place your gloved finger under the capture area to prevent any excess tissue/OCT from folding over onto your section on the capture area.

***Note:*** Given the variability in cell numbers and RNA content across different tissues, it is important to maximize tissue coverage on the capture area to achieve optimal RNA capture. In this experiment, tissue covered over 60% of the standard capture area (3 × 4 mm).

23. Store the capture area(s) tissue-side up in the cryostat until all sections have been cut.

**CRITICAL:** To prevent RNA degradation, especially when working with multiple samples, keep the tissue on the capture areas frozen inside the cryostat before starting the methanol fixation.

24. Fix the tissue by placing the capture area with the tissue section in a tube containing 1 mL of 100% Methanol (pre-cooled to −20°C).
25. Fix sections at −20°C for 30 min.

***Note:*** If intending to perform 3D alignment, take serial tissue sections. Depending on tissue type, sections up to 50–100 μm can be aligned using STIM,^14^ which also allows manual registration.

Video S3. Cryosectioning and tissue section transfer, related to “Tissue sectioning and fixation,” steps 18–22

### Hematoxylin and eosin staining and imaging

**Timing: 30 min staining, variable time imaging**

The tissue sections are stained with hematoxylin and eosin (H&E) and subsequently imaged. This later allows nuclei-based cell segmentation of the aligned imaging and transcriptomics modalities.

***Note:*** For incubations, add enough volume to cover the capture area completely. You can place the capture areas in tubes with the buffers or pipette them directly onto the areas forming a dome of liquid maintained by surface tension. For all washes, dip the capture areas in a beaker with nuclease-free water (500 mL) using forceps (Video S4).

***Note:*** Filter hematoxylin, bluing buffer and eosin Y using a 0.22 μm filter before use. Store hematoxylin and eosin Y protected from light.

26. Remove the capture areas from the cold methanol.
27. Add isopropanol to the tissue sections on the capture areas and incubate for 1 min.
28. Remove the isopropanol by tilting the capture areas onto a dust-free wipe and let the sections dry completely (∼1 min, until they look matt).
29. Add hematoxylin onto the tissue sections, and incubate for 5 min.
30. Wash the capture areas with nuclease-free water until the hematoxylin dye is completely removed (dipping 10-15×).
31. Add bluing buffer onto the tissue sections, and incubate for 2 min.
32. Wash the capture areas with nuclease-free water, dipping 5×.
33. Add buffered eosin Y onto the tissue sections, and incubate for 1 min.
34. Wash the capture areas in a beaker of nuclease-free water, dipping 10-15×.
35. Let the capture areas air-dry completely at room temperature (∼10 min).
36. Carefully remove the sticky tape using pointy tweezers before imaging.

**CRITICAL:** Be aware that scratching the flow cell surface will result in a loss of information.

37. Image the tissue on bright field using a 20× objective (Table 2).

**Table 2:** Imaging settings for the Keyence BZ-X710 microscope

Settings (specific for the Keyence BZ-X710)	
Channel	Brightfield
Camera	Color
Transmitted light	100%
Aperture Stop	20%
Oblique Illumination	OFF
Resolution/Sensitivity	High resolution
Objective lens	PlanApo_λ 20 × 0.75/1.00 mm :Default

***Note:*** If using an inverted microscope, put the capture area tissue-side-down on a coverslip for imaging (Video S5). Select a coverslip suited to your imaging system; we use a #1.5 coverslip. Clean the coverslip with RNase-away and 80% ethanol prior. The typical timing for imaging a 3 × 4 mm capture area under the listed microscope conditions is approximately 2–5 min.

38. After imaging, place the capture areas with the tissue section face up into microarray chamber wells (Video S5).

Video S4. Tissue staining with hematoxylin and eosin, related to “Hematoxylin and eosin staining and imaging,” steps 26–33

Video S5. Imaging and incubating, related to “Hematoxylin and eosin staining and imaging,” steps 36 and 37, and “Permeabilization and reverse transcription,” steps 41 and 42

### Permeabilization and reverse transcription

**Timing: 10–60 min, plus overnight incubation**

Permeabilization is performed to allow the mRNA to diffuse to and hybridize to the capture oligos. Reverse transcription covalently links the capture oligos with their spatial barcode to the captured mRNA. In Table 1 “**Permeabilization condition for tissue types tested’’**, we provide incubation times that we selected for tissue samples from Schott et al. (2024).^1^

***Note:*** We use a 100 μL reaction volume throughout the protocol for incubations in the microarray chamber.

**CRITICAL:** Ensure that the capture area is fully covered by the solutions. To prevent evaporation, use plate sealing tape to seal your microarray chamber during all following incubations in the chamber.

***Note:*** If you are unsure of the permeabilization timing for your tissues, we recommend conducting a pilot experiment using different time intervals (0, 15, 30, 45, 60 min) or varying pepsin concentrations.

***Note:*** This protocol has been tested on several other tissues from mouse (thymus, liver, inguinal white adipose tissue, triple negative breast cancer), as well as human (non-small cell lung cancer, pancreas, neuroblastoma, and melanoma).

39. Weigh and dissolve the pepsin in 2× SSC pH 2.5 to have a fresh solution of 7 U/μL pepsin.

**CRITICAL:** Do not vortex the pepsin solutions. Mix by pipetting and inverting.

40. Dilute the pepsin stock 1:10 with 2× SSC pH 2.5 to obtain a final working solution with 0.7 U/μL pepsin.
41. Prewarm permeabilization mix (0.7 U/μL) at 37°C several minutes before use.
42. Add 100 μL permeabilization mix (0.7 U/μL) per sample.
43. Seal chamber and incubate at 37°C for 45 min for the metastatic lymph node.
44. Remove the pepsin solution, carefully pipetting from the corner of the well.
45. Wash the capture area carefully with 100 μL RT Buffer once.
46. Add 100 μL RT mix per capture area.
47. Seal chamber and incubate overnight at 42°C.

**Pause point:** After overnight reverse transcription the capture areas can be washed with water 2× and stored in Tris-EDTA (TE) buffer at 4°C for 2+ days.

### Removal of single-stranded DNA by exonuclease I digestion

**Timing: ∼55 min**

Exonuclease I is used to digest single-stranded capture oligos that did not hybridize any mRNA.

48. Remove the RT mix.
49. Add 100 μL Exonuclease I mix per capture area.
50. Seal the chamber and incubate for 45 min at 37°C.

### Removing tissue from the capture areas

**Timing: 60–90 min**

These steps remove the tissue section from the capture area and denature the mRNA strand from the cDNA-mRNA hybrid.

**CRITICAL:** Keep the tissue removal mix at room temperature to avoid the precipitation of SDS. Do not vortex; mix by pipetting.

51. Remove the Exonuclease I mix.
52. Add 100 μL of 1× tissue removal mix per capture area
53. Seal the chamber and incubate for 40 min at 37°C.
54. Remove the tissue removal mix.
55. Wash as follows. The mechanical force of pipetting will remove remaining tissue.
  a. Wash the capture area with nuclease-free water three times.
  b. Wash the capture area with 100 μL of freshly prepared 0.1 N NaOH three times, incubating each wash for 5 min at room temperature.
  c. Wash the capture area with 100 μL of 0.1 M Tris (pH 7.5) three times.
  d. Wash the capture area with 100 μL of nuclease-free water three times.

***Note:*** Visually inspect the capture areas after removing the last wash to confirm complete removal of the tissue. In our experience the tissue removal is very effective. If tissue does remain, repeat the tissue removal (steps 52–55).

### Synthesis, elution, and purification of second-strand cDNA

**Timing: 3 h**

Using random priming, a second cDNA strand is synthesized from the first strand cDNA. The random priming site will later act as a unique molecular identifier.

56. Add 100 μL s strand synthesis mix per capture area.
57. Seal the chamber and incubate at 37°C for 2 h.
58. Wash with 100 μL nuclease-free water 3 times.
59. Elute the second strand product:
  **CRITICAL:** Prepare the 0.1 N NaOH from 1 N or 10 N stock freshly on the day of use.
  a. Add 100 μL of freshly prepared 0.1 N NaOH on the capture area in the chamber well, and incubate for 5 min.
  b. Recover the elution (i.e., the 2nd strand product) into a LoBind tube.
  c. Repeat steps 59 a and b, pooling the two elutions per sample.
60. Add 28.6 μL of 1M Tris-HCl pH 7.5 to each 200 μL of 2nd strand.

***Note:*** You can store the capture areas dry at −20°C as a backup of your cDNA for up to 1 year. For this, first wash each capture area twice with 100 μL of 0.1 M Tris (pH 7.5), then twice with 100 μL nuclease-free water. Let the capture areas dry before placing them in a sealed container (tube) for freezing.

61. Purify the 228.6 μL elution using AmpureXP beads at a ratio of 1.8 beads to 1× second strand product:
  a. Place the Ampure XP beads at room temperature 30 min before use.
  b. Thoroughly vortex the AmpureXP beads right before use.
  c. Add 411 μL beads to each sample. Mix by pipetting 10×.
  d. Incubate at room temperature for 10 min.
  e. Place the suspension on a magnetic tube rack for 5 min. The magnetic beads will form a pellet.
  f. Remove the clear supernatant.
  g. Keeping the tubes on the magnet, wash twice with 1 mL of 80% of EtOH, incubating for 30 s each wash. Do not disturb the bead pellet during the removal of ethanol.
    **CRITICAL:** Prepare the 80% ethanol freshly on the day of use.
  h. Keeping the tubes on the magnet, remove any residual ethanol from the tube.
    ***Note:*** You may briefly centrifuge the tubes and place them back on the magnet to collect residual ethanol.
  i. Air-dry the beads at room temperature for 5 min or until they are no longer glossy.
    ***Note:*** Avoid over-drying, visible as a cracked appearance of the bead pellet, as this may negatively affect elution.
  j. Remove the tubes from the magnetic tube rack.
  k. Add 82.5 μL nuclease-free water per sample, pipetting at least 10× to fully resuspend the beads. If necessary briefly centrifuge the tubes.
  l. Incubate at room temperature for 5 min.
  m. Place the tubes on the magnet for 30 s until the solution is clear.
  n. Collect the 82.5 μL of supernatant in a new LoBind tube. This is your cleaned up 2nd strand product.
    **Pause point:** The purified second strand can be stored at −20°C or −80°C for up to 1 year.

### qPCR for PCR cycle number assessment

**Timing: 2 h**

A qPCR is performed on the eluted second strand product to estimate the PCR cycle number required for sufficient library amplification.

62. Prepare the qPCR master mix using any p7_rev_indexing primer in the mix. Prepare enough mix for one replicate per sample and one blank control.
63. Per PCR tube mix 17.5 μL qPCR master mix and 2.5 μL of the second strand product from step 61n.
64. Run the qPCR program (Table 3).

**Table 3:** qPCR cycling conditions

Step	Temperature	Time	Cycles
Initial denaturation	95°C	3 min	1
Denaturation	95°C	30 s	40
Annealing	60°C	1 min	
Extension	72°C	1 min

***Optional:*** Include a standard melting curve program to check for amplification specificity.

**Figure 2:**
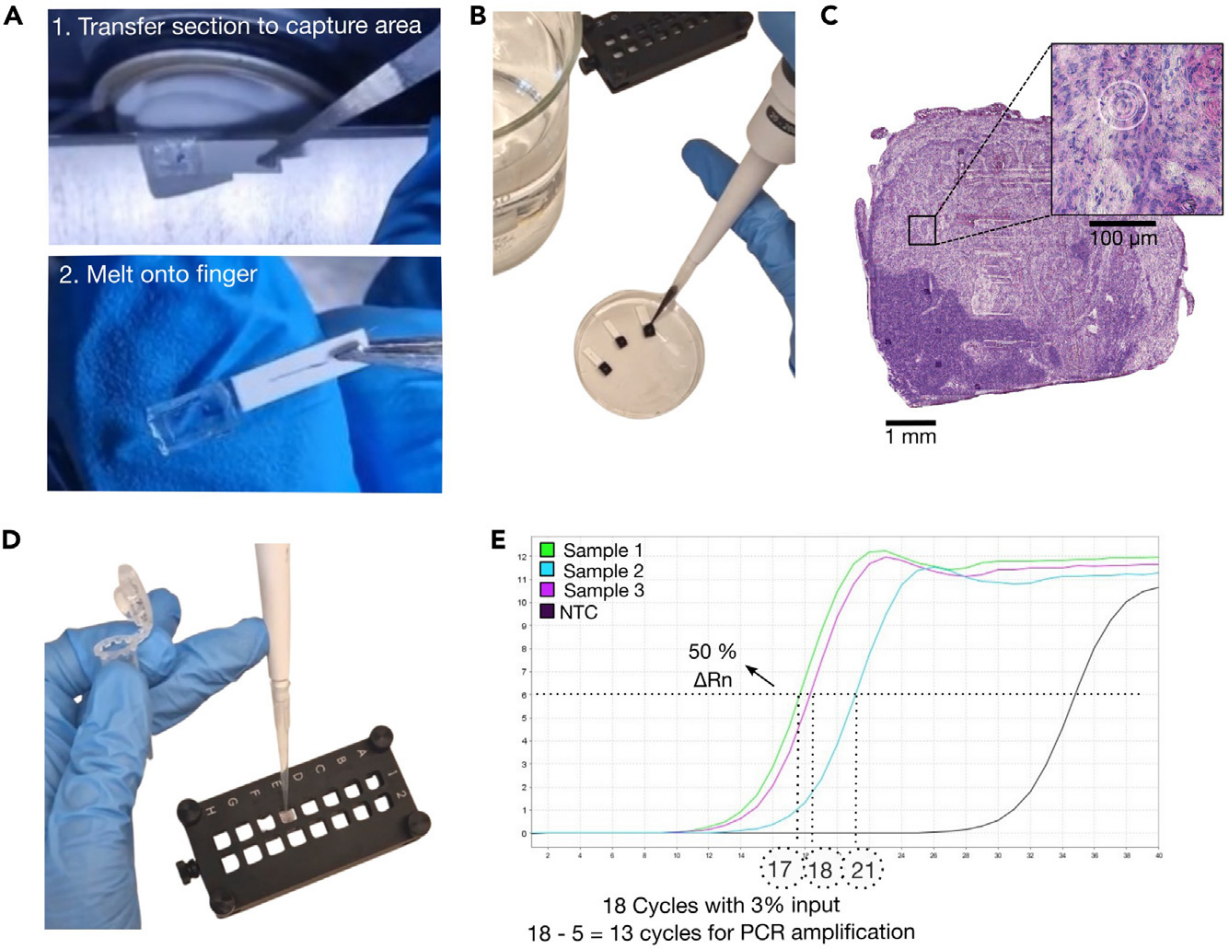
Tissue section transferral, imaging, and library preparation (A) Process of tissue placement on the capture area (here, 3 × 4 mm). (B) Setup for hematoxylin and eosin staining. (C) Stitched images of the metastatic lymph node placed on the capture area, circular marks are visible on the zoomed area. (D) Steps after imaging are performed in a microarray chamber. (E) qPCR results to determine the number of cycles for amplification, visualization with StepOne Software v.2.3 (Applied Biosystems).

***Note:*** We expect a cycling number between 11 and 14, after subtraction. Troubleshooting 4.

65. Derive the PCR cycle number required for the amplification of your sample as follows (see Figure 2E):
  a. Set a threshold at 50% of the peak ΔRn (linear scale).
  b. For each sample determine the cycle number at the intersection of the threshold and amplification curve.
  c. Subtract 5 cycles to account for the qPCR input (∼3%). This number is your recommended PCR cycle number for use in step 69.

### Library construction

**Timing: 3–4 h**

The second strand cDNA is amplified, adding sequencing adapters and an i7 index per sample. After bead purification and size selection the libraries are ready to be sequenced.

***Note:*** We use the Nextera XT Index Kit v2 Index 1 (i7) Adapters (Illumina)^4^

66. On ice, prepare the library amplification mix per sample, using a different i7 index per sample.

**CRITICAL:** Roche strongly recommends setting up the reaction on ice, due to the high proofreading activity of the KAPA enzyme leading to primer degradation at room temperature.

67. Add the remaining 80 μL 2nd strand per sample to the library amplification mix and mix well by pipetting.
68. Split each sample mix into four PCR tubes, each with 50 μL volume.
69. Run the PCR with the cycle number determined previously (step 65) (Table 4).

**Table 4:** PCR cycling conditions

Step	Temperature	Time	Cycles
Initial denaturation	95°C	3 min	1
Denaturation	95°C	30 s	(determined in step 65)
Annealing	60°C	1 min	
Extension	72°C	1 min	
Final extension	72°C	2 min	1
Hold	4°C	∞	1
70. Pool the 4 × 50 μL PCR product per sample and purify using AmpureXP beads at a 1:1 ratio of beads to PCR product.
  a. Place the Ampure XP beads at room temperature 30 min before use.
  b. Thoroughly vortex the AmpureXP beads right before use.
  c. Add 200 μL beads to each sample. Mix by pipetting 10×.
  d. Incubate at room temperature for 10 min.
  e. Place the suspension on the magnetic tube rack for 5 min. The magnetic beads will form a pellet.
  f. Remove the clear supernatant.
  g. Keeping the tubes on the magnet, wash twice with 1 mL of 80% of EtOH, incubating for 30 s each wash. Do not disturb the bead pellet during the removal of ethanol.
    **CRITICAL:** Prepare the 80% ethanol freshly on the day of use.
  h. Keeping the tubes on the magnet, remove any residual ethanol from the tube.
    ***Note:*** You may briefly centrifuge the tubes and place them back on the magnet to collect residual ethanol.
  i. Air-dry the beads at room temperature for 5 min or until they are no longer glossy.
    ***Note:*** Avoid over-drying, visible as a cracked appearance of the bead pellet, as this may negatively affect elution.
  j. Remove the tubes from the magnet.
  k. Add 20 μL nuclease-free water per sample, pipetting to fully resuspend the beads. If necessary briefly centrifuge the tubes.
  l. Incubate at room temperature for 5 min.
  m. Place the tubes on the magnet for 30 s until the solution is clear.
  n. Collect the 20 μL of supernatant in a new tube.
    ***Note:*** Optionally measure the library concentration (e.g., using the Qubit) and/or check the library profile using automated gel electrophoresis before proceeding. If the cDNA yield is low, please refer to the troubleshooting section, problem 4.
    **Pause point:** The amplified cDNA library can be stored at −20°C for up to one year.
71. Size select your sample to obtain fragments 350–1100 bp, using the Bluepippin or PippinHT 1.5% agarose gel according to the manufacturer’s instructions (https://sagescience.com/wp-content/uploads/2022/03/Quick-Guide-BDF1510-marker-R2-460048-RevB-9_07_21.pdf).

***Note:*** Alternatively manual DNA extraction from an agarose gel can be done.

72. Measure the concentration of your size-selected product; for example using the Qubit dsDNA High sensitivity (HS) quantification kit according to the manufacturer’s instructions (https://tools.thermofisher.com/content/sfs/manuals/Qubit_dsDNA_HS_Assay_UG.pdf).
73. Analyze the library profile using automated gel electrophoresis (e.g., BioAnalyzer or Tapestation), according to the manufacturer’s instructions (https://www.agilent.com/cs/library/usermanuals/public/G2938-90322_HighSensitivityDNAKit_QSG.pdf or https://www.agilent.com/cs/library/usermanuals/public/D5000_QuickGuide.pdf).

***Note:*** If your library still contains peaks, please refer to the troubleshooting section, problem 5.

**Pause point:** The size-selected cDNA library can be stored at −20°C for up to one year.

### Library sequencing

**Timing: sequencer-dependent, <1 day**

Here, we describe considerations for sequencing your final Open-ST spatial library. The general sequencing protocol will depend on sequencer used; any Illumina sequencers are compatible with the library design.

***Note:*** Choice of sequencing depth depends on the desired information content and varies with capture area size, tissue coverage and library complexity. For a ∼3 × 4 mm capture area with ∼85% tissue coverage, ∼500 M reads result in median unique molecular identifiers (UMIs) of ∼800 (ranging from 250 to ∼2500 UMIs) at a reads-over-UMIs ratio of ∼2.5, far from saturation (Figure 9). It is possible to initially sequence shallowly (∼100 million reads) to assess the library quality before sequencing deeper.

***Optional:*** We recommend quantifying your libraries for sequencing using the KAPA Library Quantification Kit from Roche.

74. Dilute, pool and denature (if required) your libraries for sequencing as detailed in the Illumina guidelines of your sequencer of choice.

**CRITICAL:** Ensure that you only pool libraries with unique indices.

75. Spike-in 1% PhiX into the pool, as detailed in the Illumina guidelines.

***Note:*** This acts as a quality control for cluster generation, sequencing, and alignment.

76. Sequence your library/pool on an Illumina sequencer, following the company’s guidelines and using the read lengths detailed in Table 5.

**Table 5:** Sequencing read lengths used for Open-ST libraries

Read	Cycles
Read 1	28–32
Index 1	8
Index 2	NA
Read2	90+

***Note:*** The optimal library loading concentration depends on the sequencer used. For the Illumina NovaSeq 6000 we obtained optimal clustering at a loading concentration of 130 pM. For the Illumina NextSeq 2000 sequencing system we recommend a loading concentration of 650 pM. For the Illumina NovaSeq X Plus we obtained optimal clustering at a loading concentration of 150 pM.

### Installation and environment setup

**Timing: 30 min**

This step sets up the necessary computational environment for running the Open-ST data processing pipeline. It includes creating a dedicated Python environment, installing the required software dependencies, and configuring the spacemake tool for read alignment and spatial mapping,^11^ and the openst tool for preprocessing of flow cell barcodes, and merging imaging with transcriptomic data.

To help users validate their setup, we provide a small example dataset consisting of three consecutive sections from the metastatic lymph node dataset (50 million reads each). This, along with all necessary auxiliary files (flow cell tile information, coordinate system) and expected outputs (h5ad files, QC reports), is available at https://github.com/rajewsky-lab/openst. Step-by-step tutorials using this dataset are provided as Jupyter notebooks at https://rajewsky-lab.github.io/openst.

77. Install the micromamba Python environment manager, and a compatible integrated development environment or text editor.

***Note:*** Refer to the installation instructions specific to your operating system (OS) (Windows, macOS, or Linux) and CPU architecture. We recommend the installation of mamba or micromamba. This can be done using the Terminal on your OS.

78. The Open-ST data processing pipeline relies on several software tools and dependencies. Download the spacemake environment file from https://raw.githubusercontent.com/rajewsky-lab/spacemake/master/environment.yaml and create a new environment by specifying this environment.yaml file:

>mamba env create -n openst -f environment.yaml

79. Activate the environment and install the openst and spacemake packages.

>mamba activate openst

>pip install openst spacemake

80. Create and navigate to a new folder where the spacemake results will be stored.
81. Download the Dropseq tools package from GitHub (https://github.com/broadinstitute/Drop-seq/releases) – for instance, you can download the file named `dropseq-3.0.0.zip`.
82. Unzip the file that has been downloaded.

***Note:*** In macOS, when using Safari, it is possible that zip files are automatically decompressed.

83. Initialize spacemake, pointing to the newly created folder after unzipping:

>spacemake init --dropseq_tools dropseq-3.0.0

84. Configure spacemake by adding the reference genomes that will be used during alignment of transcriptomic reads.

>spacemake config add_species \

 > --name species_name \

 > --reference genome \

 > --sequence /path/to/species/genome.fa \

 > --annotation /path/to/species/annotation.gtf

85. In addition to the species genome, you can add the ribosomal RNA of the species or other sequences you have spiked-in (e.g., phiX) or expect to be present in your library (e.g., a YFP reporter or viral sequences).

>spacemake config add_species \

 > --name species_name \

 > --reference rRNA \

 > --sequence /path/to/species/rRNA.fa \

 > --annotation /path/to/species/annotation_rRNA.gtf

***Note:*** For all computational steps, we recommend a Linux-based environment with at least 128 GB of RAM, 2 TB of storage, and a modern multi-core CPU (e.g., with 24 threads). Timings are given for a workstation with these properties.

### Spatial mapping of barcode sequencing

**Timing: 10 min hands-on, ∼12 h waiting**

**Figure 3:**
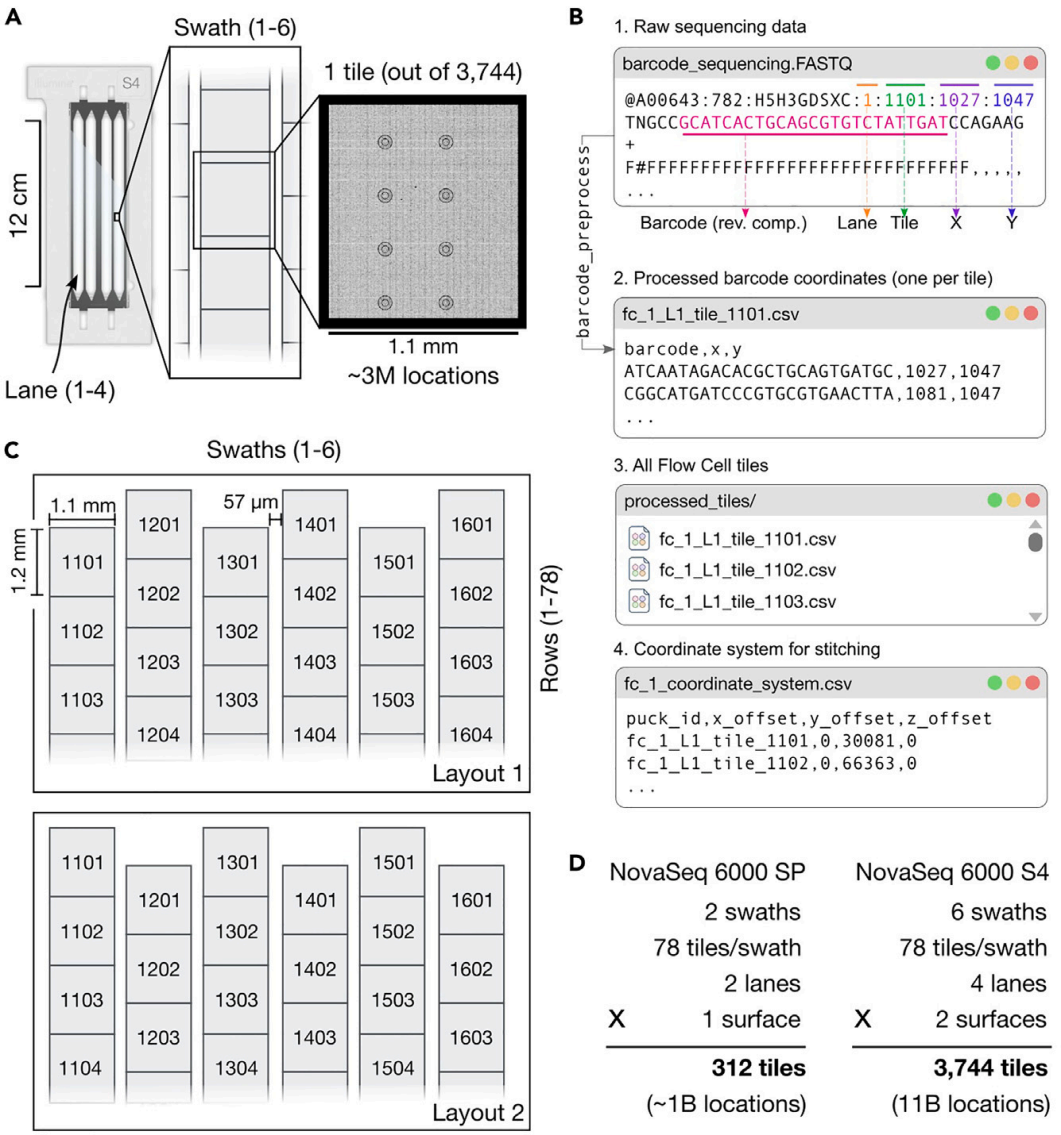
Physical organization of the spatially barcoded flow cell, and data processing workflow (A) An Illumina NovaSeq6000 S4 flow cell is depicted. We show the hierarchy of the entire flow cell, down into Lanes across surfaces, swaths, and tiles per swath. Each tile contains around 3 million valid barcoded spatial locations after first sequencing. (B) The raw sequencing data, in FASTQ format, contains the physical lane, tile ID, and relative X/Y coordinate for each barcode read, as part of the sequence identifier, as well as the barcode and its quality string. These are processed to one CSV file per tile, to map spatial barcodes to their two dimensional coordinates. All files are stored into the same directory, with standard names. A coordinate system can apply offsets at the X/Y values to each tile, converting relative into absolute coordinates. (C) Two possible layouts of tiles at a Lane (on a S4 flow cell) with respect to the offset of odd and even columns (1, 2). The global coordinates can be recovered by using a suitable coordinate system (e.g., fc_1_coordinate_system in panel B corresponds to Layout 1). (D) Calculation of number of total tiles and valid barcoded locations for NovaSeq 6000 SP and NovaSeq 6000 S4 flow cells.

Here, we generate the barcode-to-coordinate map: for each flow cell, these plain text files have three columns: barcode, x_position and y_position (Figure 3B). These are later used by spacemake to reconstruct the spatial coordinates from transcriptomic libraries. This process is performed only once per barcoded flow cell.

86. Create the barcode-to-coordinate map for all tiles. Make sure to specify the arguments --crop-seq to a compatible Python slice (e.g., 5:30 will take from the 6th to the 30th nucleotide from the reads), and --rev-comp to store the reverse complement.

>openst flowcell_map \

 > --bcl-in /path/to/fc/bcl \

 > --tiles-out /path/to/fc_tiles \

 > --crop-seq 5:30 \ # for default Open-ST sequencing recipe

 > --rev-comp

This will create as many barcode-to-coordinate compressed text files as tiles in the flow cell.

***Note:*** While BCL files are recommended for optimal processing speed through parallel demultiplexing, the flowcell_map script also accepts fastq files as input. However, when starting from fastq files, the decompression process cannot be parallelized, which may increase processing time during the initial barcode extraction steps. In this case, replace --bcl-in /path/to/fc/bcl by --bcl-in /path/to/fc/barcodes_1.fastq.gz /path/to/fc/barcodes_1.fastq.gz …

**CRITICAL:** Either bcl2fastq or bclconvert are required for openst flowcell_map. Please install them following instructions from Illumina, and make them available via the PATH environment variable. For instance, in Linux: export PATH = /path/to/bcl2fastq:$PATH

***Note:*** This step is storage-intensive, especially with S4 flow cells. Raw sequencing reads, in either FASTQ or BCL format, are 100–300GB in size. Another 2 TB of disk space are required to store the intermediate results of openst flowcell_map, which are cleared after processing, and ∼100 GB required for the final collection of tile files, which are kept for all subsequent steps.

***Note:*** The number of individual tiles you obtain from sequencing depends on the flow cell type and the number of lanes used, ranging from 468 tiles (for SP flow cells) to 3,744 tiles (for S4 flow cells) (Figure 3A). Initially, each tile has its own set of relative spatial coordinates. If we were to simply combine these tiles, their (x, y) coordinates would overlap. To solve this, we need to 'stitch' the tiles together, converting their relative coordinates into a single set of absolute coordinates that cover the entire flow cell. This stitching process can be done automatically using either 'spacemake' or 'openst' (see Steps 88 or 93). To perform the stitching, you need to provide a coordinate system file that specifies the correct position of each tile relative to all others in the capture area (Figures 3B and 3C). Different flow cells might require different coordinate systems, as the relative positioning of tiles might change (Figure 3C). Spacemake includes one such coordinate system by default, and scripts to create custom coordinate systems for NovaSeq 6000 flow cells (S4 by default, all other supported).

### Processing of transcriptomic reads

**Timing: 15 min hands-on, ∼15 h waiting (per 500 M reads)**

After Step 76, the raw transcriptomic reads need to be preprocessed and mapped to the reference genome. Also, reads are spatially mapped against the entire flow cell to identify the tiles within the capture area used, thus it is not necessary to keep track of the capture area origin on the flow cell.

***Note:*** In the Terminal, make sure that the current directory is the same as the one where spacemake was initialized (see Step 80). Also, make sure that the openst environment is activated, i.e., micromamba activate openst

***Note:*** Estimated times are provided for spatial mapping of a NovaSeq6000 S4 flow cell, and a single sample of 3 × 4 mm physical size (∼12 tiles) sequenced at ∼500 million to 1 billion reads.

87. If your sequencing provider shared basecalls and not FASTQ files, demultiplex the sequencing of the Open-ST library.

>bcl2fastq \

 > --no-lane-splitting \

 > --runfolder-dir /path/to/bcl/files \

 > -o /path/to/demux \

 > --sample-sheet /path/to/sample_sheet.csv

88. Add the sample(s) to spacemake using the following command template:

>spacemake projects add_sample \

 > --project_id project_id \

 > --sample_id sample_id \

 > --R1 /path/to/demux/sample_id_R1.fastq.gz \

 > --R2 /path/to/demux/sample_id_R2.fastq.gz \

 > --species species_name \

 > --puck openst \

 > --run_mode openst \

 > --barcode_flavor openst \

 > --puck_barcode_file /path/to/fc_tiles/∗.txt.gz \

 > --map_strategy STAR:genome:final

***Note:*** With the --barcode_flavor, --puck and --run_mode that ship with spacemake, it will generate individual (h5ad) files per sample and tile, as well as a single, unified (h5ad) file per sample that contains all tiles. For the last output, a coordinate system is used (Figures 3B and 3C).

***Note:*** The --map_strategy parameter defines the sequence of alignment steps. In this example, "STAR:genome:final" means “align to the genome using STAR, and designate the output as the final BAM file”. More complex strategies can be specified, in case additional genome sequences were previously provided, such as "bowtie2:rRNA->STAR:genome:final" for sequential alignment, or "bowtie2:rRNA,STAR:genome:final" for parallel alignment. References not flagged as “final” are not included in digital gene expression (DGE) files.

89. Repeat Step 88 with different project_id and sample_id to add multiple samples. When spacemake is started, it will automatically process all added samples in parallel.

***Note:*** If a sample has been re-sequenced at a higher depth to obtain higher molecular counts, you can merge the individual samples by using spacemake projects merge_samples.

90. Start spacemake. Spacemake automatically performs quality control, read preprocessing, alignment, mapping to spatial locations, and gene quantification.

>spacemake run --cores 16 --keep-going

***Note:*** Make sure to adapt the number of cores depending on your machine. Spacemake requires at least 4 cores.

**CRITICAL:** Adding the --keep-going flag allows spacemake to keep running in case individual jobs fail, e.g., from one of the samples in the run, while letting the others finish. In case individual jobs fail and you need to restart the pipeline, make sure you add the --rerun-incomplete flag, which will attempt to rerun and complete these.

91. After the pipeline completes, review the automatically generated quality control and analysis reports (see Troubleshooting 9 and 10).
  a. QC reports can be found at: projects/project_id/processed_data/sample_id/illumina/complete_data/qc_sheets
  b. Automated analysis can be found at: projects/project_id/processed_data/sample_id/illumina/complete_data/automated_analysis
  c. Files that contain the digital gene expression (DGEs) can be found at: projects/project_id/processed_data/sample_id/illumina/complete_data/dge

**Figure 4:**
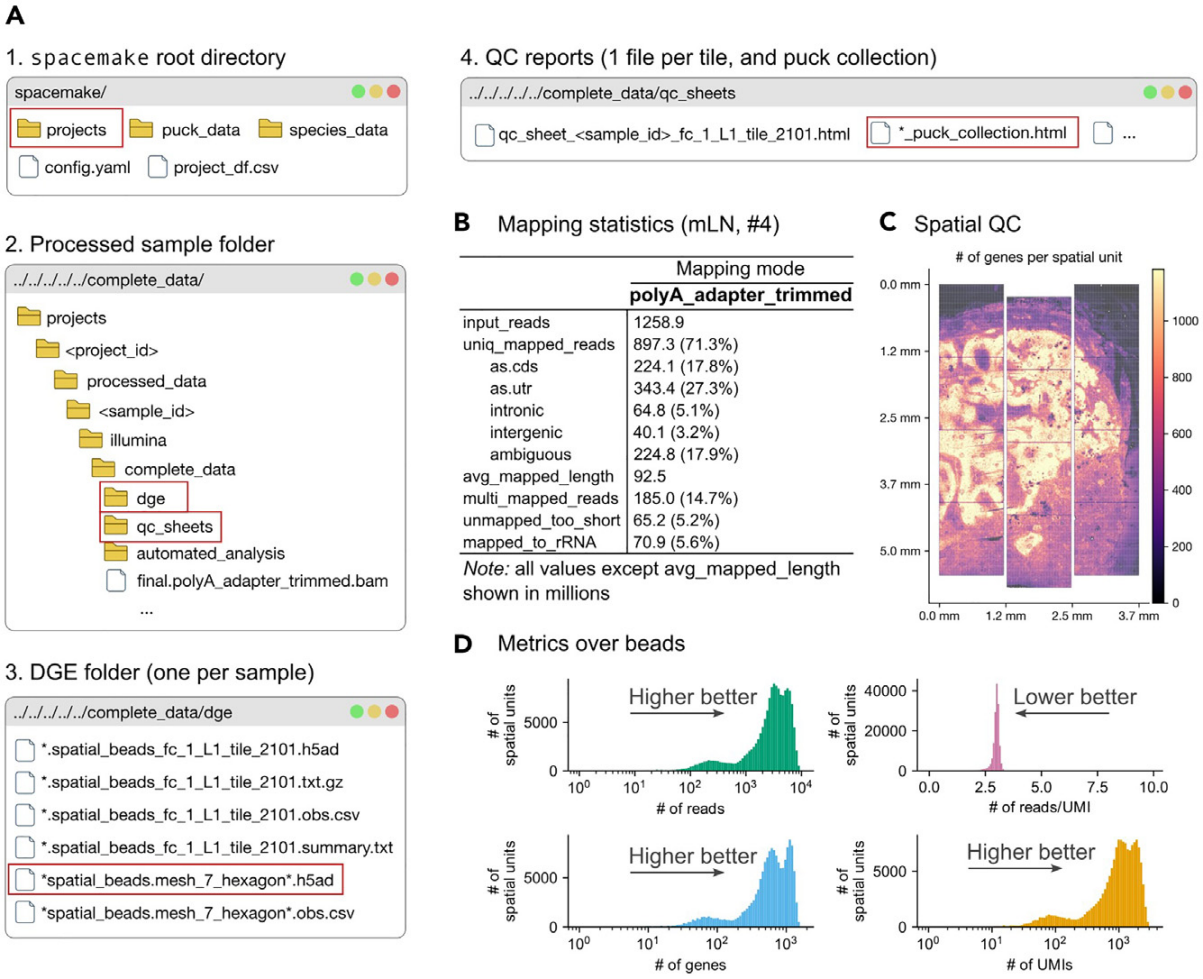
File structure after running spacemake and first assessment of quality of datasets (A) Walkthrough of the folder structure from the spacemake root directory to two key files: the h5ad object containing the spatial cell-by-gene expression matrix, and the QC report in HTML format. (B) Mapping statistics for a high quality sample. For samples sequenced at similar depth (∼500 M), these numbers are representative of the capabilities of Open-ST. Different values (especially, lower uniquely mapping and higher rRNA), might indicate issues with the tissue. (C) Visual validation of spatial mapping. Shown is a high quality sample where, as expected, reads per UMI are uniform in space, and UMI density distinguishes tissue from background. (D) Metrics over beads (in this case, each from the default 7 μm-side hexagonal grid). Shown is a “gold standard”, i.e., these metrics are expected for a sample with high quality. In particular, PCR bias (# of reads / UMIs) values should tend to 1; lower values are indicative of more complex libraries (desired). The QC reports show all distributions for 7 μm-side hexagons, without filtering, i.e., all *pseudocells* are included regardless of being in or out of the tissue. These are useful for assessing the background levels (outside of tissue) across all metrics. mLN: human metastatic lymph node.

***Note:*** The output of spacemake is organized into two main categories: non-meshed files (original 0.6 μm resolution) and meshed files (aggregated into ∼7 μm-side hexagons, by default) (see Figure 4A for file structure). The 'puck collection' files refer to those where all tiles have been stitched into a single view of the sample. For quick QC, we recommend examining the meshed objects, as their size approximates individual cells, making quality assessment easier. The QC reports folder contains visualizations and metrics for sample quality evaluation (Figures 4B–4D), while the automated analysis folder provides clustering and differential expression results. Importantly, the QC report includes all spatial units (7 μm-side hexagons) without filtering, including those from background areas. Consequently, median values and distributions in these reports are skewed towards lower values. This unfiltered approach allows for a comprehensive view of the data but requires careful interpretation, particularly when assessing gene detection rates and UMI counts across the entire capture area.

***Note:*** The units of the spatial coordinates from any h5ad object created by spacemake might not be calibrated by μm/pixel. For accurate measurement of physical distances, proceed with the alignment to the image as described below.

### Combining imaging and transcriptomics

**Timing: 30 min hands-on time, 20 min waiting**

This step integrates the stitched microscopy images with the spatial transcriptomics data, creating a unified dataset for analysis. It includes spatial stitching of individual microscopy imaging tiles, merging imaging and transcriptomics modalities, and performing pairwise alignment between the imaging and transcriptomic data to enable accurate spatial mapping of gene expression.

92. Prepare stitched imaging data.

***Note:*** Ensure your microscopy images are stitched into a single image (in TIFF format) before proceeding. The method for this may vary depending on your microscope setup. Refer to the Open-ST website for more detailed instructions depending on the microscope. We also recommend referring to the ImageJ tutorial for the “Grid/Collection Stitching” plugin.

***Note:*** If the image is generated outside of the openst pipeline, make sure it is in TIFF format (.tif extension), and copy it to the following location as “Image_Stitched_Composite.tif”: projects/project_id/processed_data/sample_id/images/Image_Stitched_Composite.tif.

93. Combine individual tile data into a single file with correct spatial offsets (Figures 3C and 4C):

>openst from_spacemake

 > --project-id project_id \

 > --sample-id sample_id \

 > spatial_stitch

***Note:*** This and the following commands should be executed from within the same folder where spacemake was executed. This way, openst makes use of the spacemake’s data structure, and automatically identifies the relevant input files, thus simplifying the commands.

***Note:*** If you configured the sample in spacemake with a run_mode with mesh_data: False, you do not need to run this step.

94. Integrate the image data into the h5ad object containing the transcriptomic data:

>openst from_spacemake \

 > --project-id project_id \

 > --sample-id sample_id \

 > merge_modalities

95. Perform pairwise alignment between the two modalities. This enables the accurate mapping of gene expression data onto the tissue image.

>openst from_spacemake \

 > --project-id project_id \

 > --sample-id sample_id \

 > pairwise_aligner

***Note:*** You can specify the argument --device cuda to use a compatible GPU accelerator if it is available, which will reduce the alignment time significantly.

***Note:*** There are several user-modifiable parameters that can be changed to improve the quality of pairwise alignment depending on the type of sample (Table 6).

**CRITICAL:** if the automatic alignment fails, proceed to Step 96, and perform the two rounds of alignment (coarse and fine) manually, or check Troubleshooting 6.

**Figure 5:**
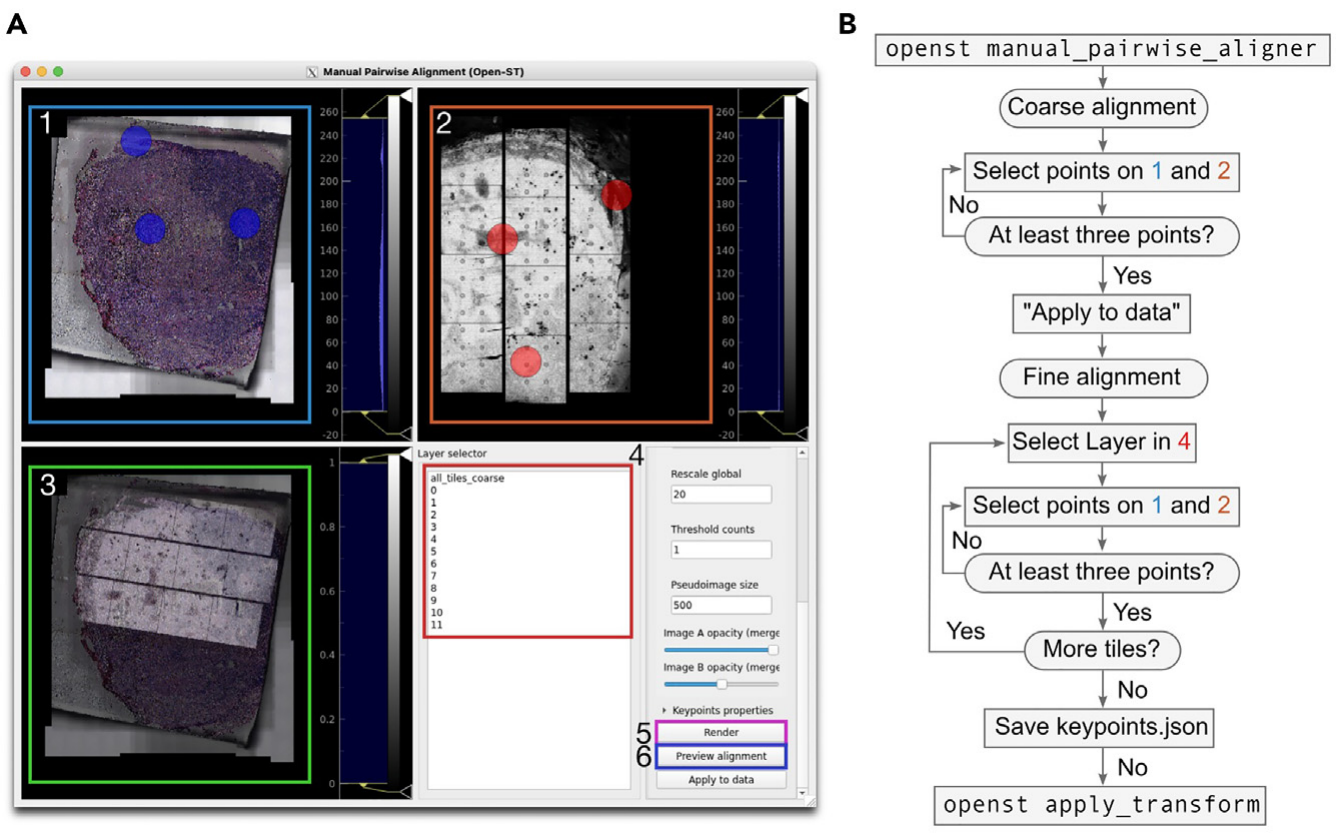
Manual Pairwise Alignment Graphical User Interface (GUI) for Open-ST data (A) The graphical user interface for manual alignment of imaging and spatial transcriptomics data. (1) Imaging modality view, showing the tissue section with manually selected corresponding visual landmarks (blue circles). Visual landmarks can be any two similar areas of tissue morphology or, more specifically, the small fiducial circles visible across both images (see Figure 2C). (2) Spatial transcriptomics modality view, displaying gene expression data with corresponding selected keypoints (red circles). (3) Overlay view of both modalities for alignment verification, after applying “Preview alignment”. (4) Layer selector for individual tile selection during fine alignment. (5) 'Render' button to update views after parameter changes. (6) 'Preview alignment' button for testing and confirming alignments. Not highlighted: 'Keypoints properties' dropdown menu for saving and loading keypoint data, and sliders for adjusting image opacity in the overlay view. (B) Workflow diagram for the manual alignment process, using the command-line application (first and last steps), and the GUI.

96. Visualize and explore the results of pairwise alignment with the GUI (see Figure 5):

>openst from_spacemake \

 > --project-id project_id \

 > --sample-id sample_id \

 > manual_pairwise_aligner

97. In the GUI, select the spatial coordinates layer under “Data properties”->”Spatial coordinates” (e.g., obsm/spatial_aligned_coarse), and the image data under “Image data”->”...” (e.g., uns/spatial/staining_image).
98. Select “all_tiles_coarse” to visualize the whole collection of tiles, or one of the numbers to select a specific tile (close-up visualization).
99. Click “Render” to display the specified spatial coordinates and image for the selected layer. Diagnose the alignment visually, relying on common features between the modalities, and the fiducial circles visible across the two modalities.
100. If necessary, refine the results via manually setting corresponding points.
  a. If the alignment of “all_tiles_coarse” looks reasonable, skip to (e).
  b. To perform coarse alignment, select the “all_tiles_coarse” layer, then “Render”, and double-click to select at least three corresponding points on Image 1 (1 in Figure 5A) and Image 2 (2 in Figure 5A). You might need to select the checkbox “Is unaligned data” under “Rendering settings”.
  c. Click 'Preview alignment' (6 in Figure 5A) to check the result.
  d. If satisfactory, click 'Apply to data' (6 in Figure 5A) to save the coarse alignment.
  e. Use the Layer selector (4 in Figure 5A) to choose individual tiles. For each tile, double-click to select at least three corresponding points on Image 1 and Image 2. Click 'Preview alignment' to verify. Repeat for all tiles.
  f. Open the 'Keypoints properties' dropdown menu, and click 'Save keypoints' to save the current selection as 'keypoints.json'.
  g. Apply Final Transformation:

>openst apply_transform --keypoints-in keypoints.json

***Note:*** Use the 'Render' button (5 in Figure 5A) to update views after parameter changes. Adjust image opacity using sliders for better overlay visualization (3 in Figure 5A). The protocol can be repeated by adapting the “Data properties”->”Spatial coordinates” iteratively, if further refinement is needed.

***Note:*** the spatial coordinates in the stitched and aligned h5ad file are in pixel units, not physical units (i.e., μm). To convert to μm, multiply the spatial coordinates (both X and Y axes) by the μm/pixel factor. For instance, for the 20× magnification images we obtained, the conversion factor is 0.345 μm/pixel (multiplicative). This can be done separately with scanpy.

**Table 6:** User-modifiable parameters in “openst pairwise_aligner”

Parameter	Default	Description
--rescale-factor-coarse	20	Rescaling factor for input image during coarse alignment. Lower values increase precision but computational cost.
--threshold-counts-coarse	1	Minimum count threshold for spatial coordinates in coarse alignment. Higher values may improve signal-to-noise ratio.
--pseudoimage-size-coarse	500	Size (pixels) of pseudoimage in coarse alignment. Larger sizes may increase precision but computational cost.
--ransac-coarse-residual-threshold	2	RANSAC residual threshold for coarse registration. Lower values increase alignment strictness.
--ransac-coarse-max-trials	2	Number of RANSAC iterations (x1000) in coarse registration. Higher values may improve alignment at cost of time.
--rescale-factor-fine	10	Rescaling factor for input image during fine alignment. Lower values increase precision but computational cost.
--fine-registration-gaussian-sigma	2	Gaussian blur sigma for fine registration. Higher values smooth out noise but may lose fine details.
--threshold-counts-fine	0	Minimum count threshold for spatial coordinates in fine alignment. Higher values may improve signal-to-noise ratio.
--pseudoimage-size-fine	2000	Size (pixels) of pseudoimage in fine alignment. Larger sizes may increase precision but computational cost.
--ransac-fine-residual-threshold	2	RANSAC residual threshold for fine registration. Lower values increase alignment strictness.
--ransac-fine-max-trials	2	Number of RANSAC iterations (x1000) in fine registration. Higher values may improve alignment at cost of time.
--fine-min-matches	50	Minimum number of matching keypoints in fine alignment. Higher values ensure more robust alignment but may fail in low-quality data.

### Cell segmentation and single-cell quantification

**Timing: 30 min**

This step involves segmenting individual cells from the aligned imaging data and assigning transcripts to these segmented cells. It creates a cell-by-gene expression matrix that integrates spatial information with transcriptomic data, enabling single-cell level analysis within the tissue context.

101. Perform cell segmentation on the aligned imaging data:

>openst from_spacemake \

 > --project-id project_id \

 > --sample-id sample_id \

 > segment \

 > --model HE_cellpose_rajewsky

***Note:*** You can specify the argument --device cuda to use a compatible GPU accelerator if it is available, which will reduce the alignment time significantly.

***Optional:*** Depending on the imaging modality, you might want to select different models or segmentation parameters, explained in Table 7 (see Troubleshooting 8).

***Note:*** Alternative cell segmentation tools such as QuPath or ImageJ can be integrated into the Open-ST pipeline. Users can import segmentation masks from these tools using the openst merge_modalities command and proceed with the standard workflow.

**Figure 6:**
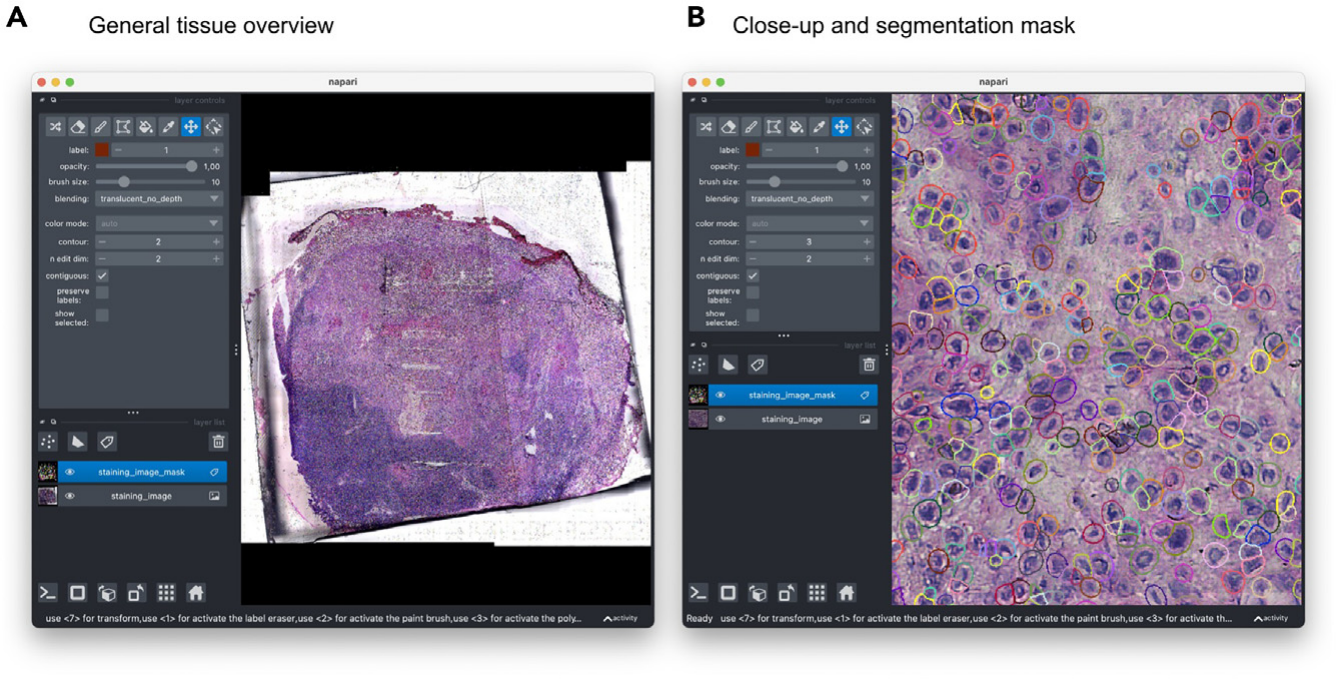
Interactive visualization and quality assessment of cell segmentation with Napari (A) General tissue overview showing the Napari interface displaying two image layers: the H&E staining of a metastatic lymph node (bottom layer) and the corresponding segmentation mask generated by the openst segment tool (top layer). (B) Close-up view and segmentation mask of a specific region, demonstrating the high-resolution cell segmentation achieved by Open-ST. The colorful pixelated overlay represents individual segmented cells, allowing for detailed manual validation of segmentation quality.

102. Visually assess the segmentation results (Figure 6):

>openst from_spacemake \

 > --project-id project_id \

 > --sample-id sample_id \

 > preview \

 > --image-keys

uns/spatial/staining_image uns/spatial/staining_image_mask

103. If you are not satisfied by the cell segmentation result, you can try again with other parameters or models (see Troubleshooting 7), and proceed again from Step 101.
104. Create a single h5ad file containing the cell-by-gene matrix based on the segmented cells

>openst from_spacemake \

 > --project-id project_id \

 > --sample-id sample_id \

 > transcript_assign \

 > --spatial-key

obsm/spatial_manual_fine \

 > --mask-in

uns/spatial/staining_image_mask

***Note:*** Make sure that --spatial-key is set to the name of the best pairwise-aligned spatial coordinates generated either via (semi)automatic or manual segmentation. You can always check the available layers in the h5ad file from the terminal with the command:

>openst from_spacemake \

 > --project-id project_id \

 > --sample-id sample_id \

 > preview \

 > --file-structure

105. A new file containing the segmented cell-by-gene matrix, in h5ad format,^19^ is available under: projects/project_id/processed_data/sample_id/multimodal/stitched_segmented.h5ad

**Table 7:** User-modifiable parameters in “openst segment”

Parameter	Default	Description
--flow-threshold	0.5	Cellpose’s 'flow_threshold' parameter. Higher values result in fewer cell detections.
--cellprob-threshold	0	Cellpose’s 'cellprob_threshold' parameter. Higher values result in stricter cell detection.
--diameter	20	Cellpose’s 'diameter' parameter. Adjust based on average cell size in pixels.
--dilate-px	10	Number of pixels to extend the outlines of the segmentation mask.
--outline-px	0	Width of object outlines in pixels (only if > 0).
--tissue-masking-gaussian-sigma	5	Gaussian blur sigma used for isolating tissue on the staining image.

### 3D reconstruction into a virtual tissue block from serial sections

**Timing: 10 min hands-on, 10 min processing (for 3 sections)**

This step integrates multiple 2D Open-ST datasets into a cohesive 3D representation, creating a virtual tissue block. It involves aligning serial tissue sections using STIM (Spatial Transcriptomics as Images) software,^14^ which enables the reconstruction of 3D gene expression patterns and tissue architecture from individual 2D datasets.

106. Install STIM via mamba (Windows, macOS and Linux are supported).

>mamba create -n stim -c conda-forge -c bioconda stim

>mamba activate stim

107. Create a container-dataset containing the individual segmented slices – in this example, we assume that 3 sections are available:

> st-add-slice -c openst_3d_example.n5 -i

/path/to/sample_1/stitched_segmented.h5ad

>st-add-slice -c openst_3d_example.n5 -i

/path/to/sample_2/stitched_segmented.h5ad

>st-add-slice -c openst_3d_example.n5 -i

/path/to/sample_3/stitched_segmented.h5ad

108. Perform pairwise alignment of pairs of slices:

>st-align-pairs -c openst_3d_example.n5 -n 15 --maxEpsilon 0 --range 2 --scale 0.03

109. Perform the global alignment of all slices and compute the final transformations:

>st-align-global -c openst_3d_example.n5 --skipICP

***Note:*** before converting back to an h5ad object, assess the quality of 3D alignment visually. This can be done with GUI tools from STIM, i.e., st-bdv-view, st-bdv-view3d, or st-align-interactive to interactively refine the alignment based on visual landmarks.

110. Use the from_3d_registration utility in the openst package to convert the STIM container to a single h5ad object. This leverages the stimwrap Python bindings for STIM. This can be parameterized to also apply the coordinate transformation to the staining images.

## Expected outcomes

**Figure 7:**
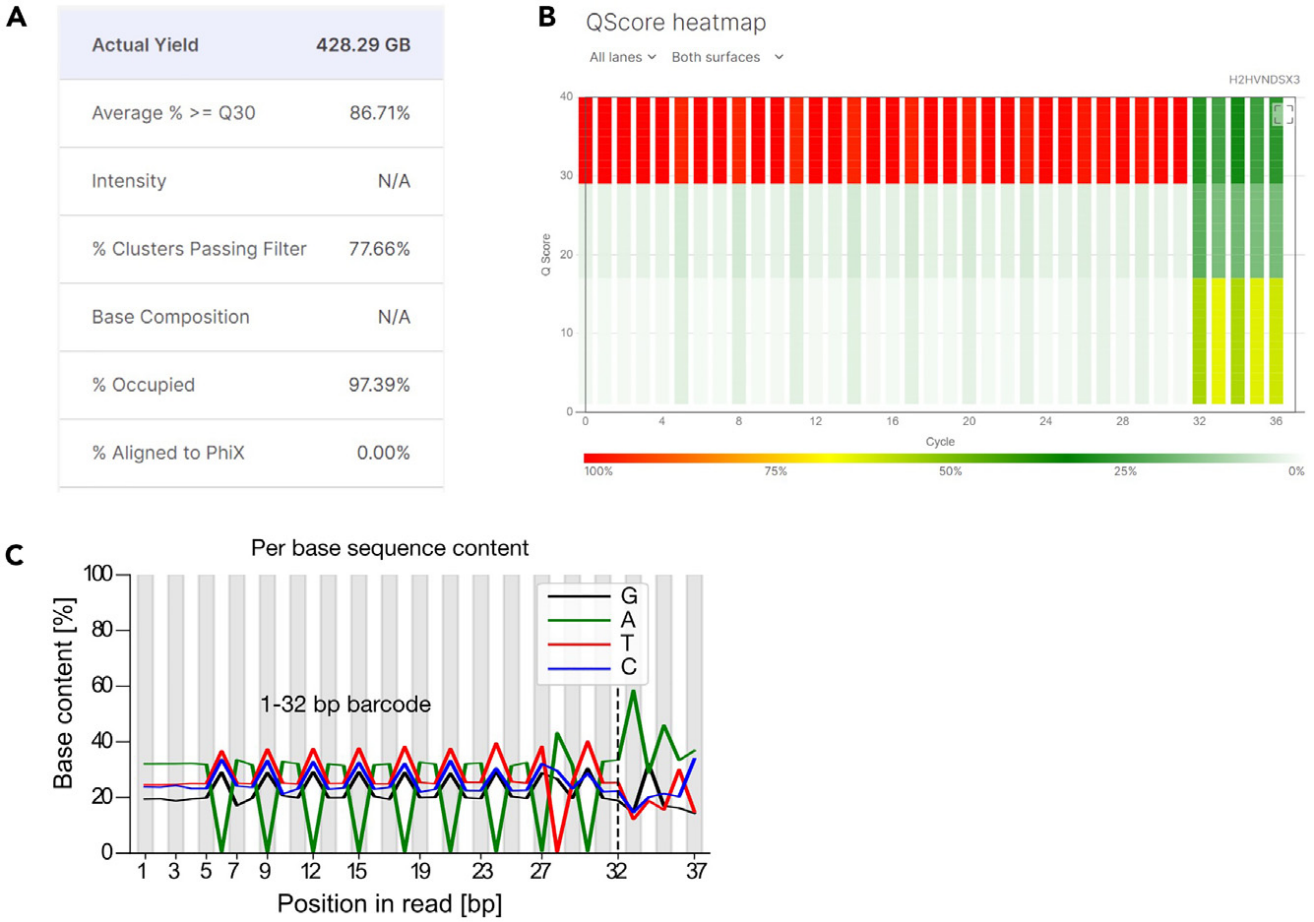
Quality metrics of a barcoded Illumina NovaSeq 6000 S4 flow cell (A) Raw and passing filter (PF) cluster density per lane; from the Illumina Sequencing Analysis Viewer (SAV, v.3.0.0). (B) Heatmap of sequencing quality score (Q score) per cycle from the Illumina SAV. >Q30 indicates an inferred base call quality of >99.9%. (C) Barcode base content (%) per sequencing cycle.

Sequencing of the synthetic barcode library for Open-ST capture area generation is expected to yield a flow cell with 85% ≥ Q30, ≥ 73% passing filter and ∼97% clusters occupied. The base composition of the barcode is expected to follow the pattern of the HDMI32 oligo (NNNNNBNNBNNBNNBNNBNNBNNBNNBVNBNNA) designed by Cho et al. (2021).^3^ QC metrics of an example flow cell are shown in Figure 7. Request your sequencing provider to deliver the data as basecall files.

**Figure 8:**
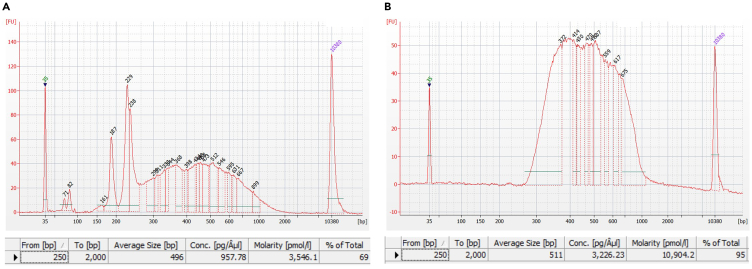
Open-ST library profiles before and after size selection (A) Library profile of a metastatic lymph node library before size selection. (B) As (A), but after gel-based size selection. BioAnalyzer traces visualized with 2100 Expert software version B.02.10.SI764 (Agilent) are shown. Total cDNA amount and average size of the region of interest are indicated in the table below.

In your H&E image of the tissue on the capture area, the circular alignment marks should be on one focus plane with the tissue (see Figure 2C). From the qPCR we expect to define a cycling number between 11 and 14. The PCR reaction is expected to yield 2–12 ng/μL (in the 20 μL from step 70n), varying with tissue coverage, RNA quality, permeabilization efficiency or overall tissue RNA content. Before size selection artifact peaks at ∼185 bp and 226 bp can be observed, with the majority of the sample comprising the library peaking at ∼500–600 bp (Figure 8). The expected yield after size selection is 50–80% of the concentration in the size range of interest (350–1100 bp), as described in the specifications of the PippinHT instrument (https://sagescience.com/support/pippinht-faqs/). After size selection, all fragments under 250 bp should be completely removed, with the library profile appearing smooth (Figure 8).

Sequencing data of your Open-ST library should match the expected QC values and output indicated by Illumina for the respective sequencer used. Request already demultiplexed files from your sequencing provider.

**Figure 9:**
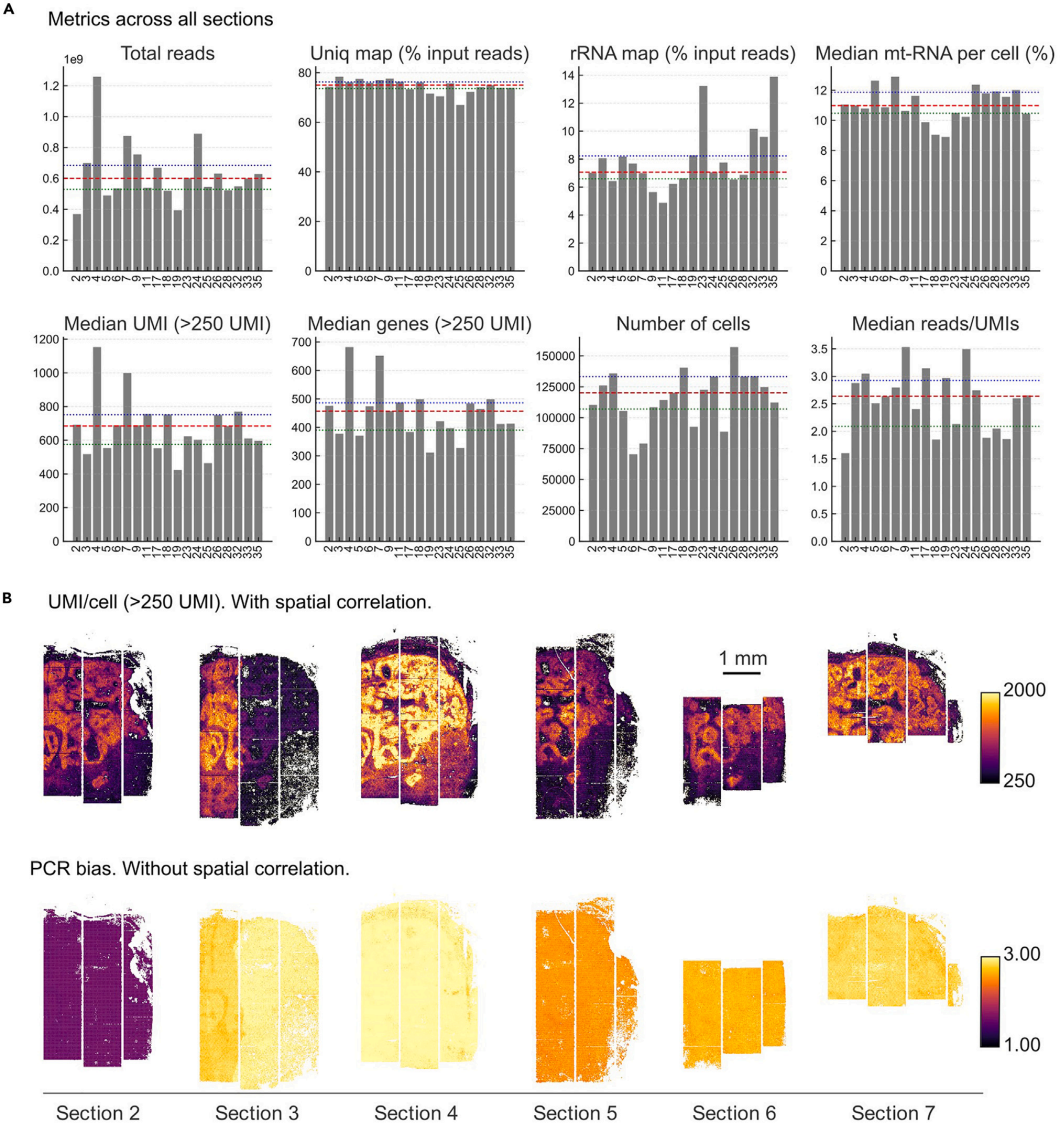
Quality control assessment of Open-ST data from metastatic lymph node sections (A) Barplots displaying key metrics across all processed sections, including total reads, uniquely mapped reads, rRNA mapped reads, median mt-RNA percentage per cell, number of cells, median PCR bias, median UMI, and median genes detected. Red dashed lines indicate median values, while blue and green dotted lines represent the first (Q1) and third (Q3) quartiles, respectively. (B) Spatial plots of UMI per cell (top row) and PCR bias (bottom row) for six representative sections. Here, a regular grid of 7 μm hexagons is used instead of cell segmentation for the first QC of the data. Only cells passing a cutoff of 250 UMIs are displayed. UMI counts show clear spatial correlation with tissue structure, while PCR bias remains relatively uniform across the tissue, as expected for high-quality data. Color scales are consistent across all sections for each metric, facilitating direct comparisons. In (B), only the first 60 μm across the Z-axis (out of 350 μm) are shown for illustrative purposes. Note that some spatial artifacts are visible, such as higher UMI content in one swath of section #3, and incomplete coverage in sections 6-7. These issues were identified in this early experiment, leading to the discovery of 1.5 cm blind spots at the flow cell ends, which are now accounted for in the protocol.

Finally, assess the QC reports created by spacemake (Step 91). Look at these prior to continuing with the rest of the alignment and segmentation pipeline. When reviewing, look for consistent gene and UMI counts across the tissue, clear tissue-background separation, and absence of spatial artifacts. Successful samples typically show high gene detection (absolute number will depend on the sequencing depth), low PCR duplication (as close as possible to 1, values beyond 5 might indicate sequencing saturation), and clear biological structures (see Figure 9 for examples of expected QC results across sections, which also highlights the reproducibility). Across sections of one tissue, mapping statistics are consistent (around 75% of uniquely mapping across sections, around 7% of rRNA content, and around 11% of mt-RNA per cell), as well as the number of cells per section, their PCR bias (# of reads / UMIs), number of UMIs and number of genes per hexagonal unit (here assumed “per cell”). Some spatial artifacts are noticeable, like one swath of section #3 having higher UMI content than the rest. Also, sections 6-7 contain transcriptomic information only for half of the tissue section (Figure 9B), caused by the unimaged ends of the flow cell not being removed (we identified these blind areas upon this experiment). When satisfied with the outcomes from these preliminary QC reports, proceed with pairwise alignment, segmentation, and 3D alignment of the virtual tissue block. Intermediate steps may be judged visually, e.g., by manually assessing the quality of the segmentation mask, pairwise alignment and 3D registration. Finally, the aligned and segmented cell-by-gene h5ad objects can be judged similarly as the preliminary QC reports: look for consistent gene and UMI counts, absence of artifacts, *etc*..

## Quantification and statistical analysis

**Figure 10:**
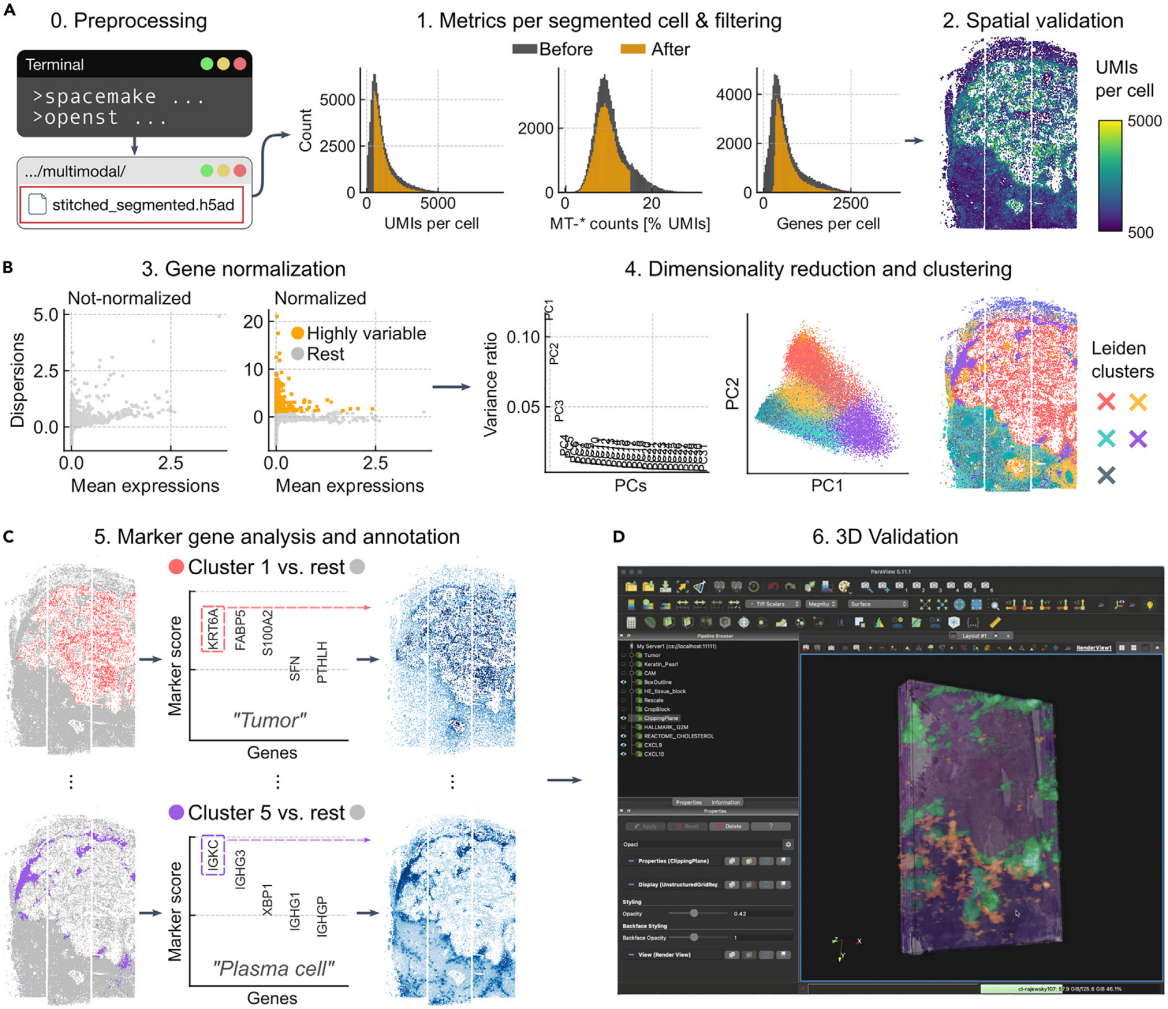
Typical downstream analysis workflow for Open-ST data (A) Initial data processing steps. The analysis begins with the stitched_segmented.h5ad file, which undergoes metrics calculation per segmented cell and filtering, followed by spatial validation of the data. (B) Core analysis pipeline. The workflow continues with gene normalization, dimensionality reduction, and clustering of the data to yield cell types (or regions) in space. (C) After clustering, marker gene analysis is performed, including ranking plots and spatial validation of gene expression patterns, which help with the annotation of expression clusters, and lead to the identification of potentially novel disease-relevant candidate genes and gene programs, in space. (D) Using ParaView^6^ for 3D visualization of spatial gene expression in the histology context allows the validation of the annotations and their spatial patterns.

The above spacemake and openst pipeline generates the objects which contain the cell-by-gene expression matrix and cell spatial locations, per section. These can be further analyzed to reveal cellular heterogeneity, identify marker genes, and visualize transcriptomic patterns within their spatial context, among other downstream results (Figure 10). This section outlines the analysis pipeline using widely adopted tools and techniques. Here, we outline the basic workflow used to analyze a metastatic lymph node Open-ST dataset.^1^

We use scanpy,^12^ a Python-based toolkit for analyzing single-cell gene expression data, which provides comprehensive methods for preprocessing, clustering, differential gene expression analysis, and visualization. Additionally, in the case of 3D reconstructions from serial sections, we utilize ParaView,^6^ a powerful open-source software for 3D data visualization, to create interactive 3D representations of tissue histology and gene expression patterns.

This exploratory analysis workflow consists of several key steps: data preprocessing, gene expression normalization, dimensionality reduction, clustering, marker gene identification, spatial gene expression analysis, and 3D visualization (Figure 10). Data preprocessing involves filtering the cells with too low or too high counts and genes, by assessing the distribution of unique counts and genes per cell (Figure 10A); then, the counts at the remaining cells are normalized to the depth per cell with a correction factor for the median depth across cells, and log-transformed, to account for technical variations. Dimensionality reduction techniques such as PCA are applied to facilitate unsupervised clustering, e.g., via the Leiden algorithm,^20^ to identify distinct cell populations based on their gene expression profiles. Currently, the above steps are adapted from the common practice for single-cell RNA-sequencing analysis^21^^,^^22^ (see limitations). Further spatial analysis such as joint analysis with imaging data, discovery of spatially variable genes, cellular neighborhood analysis, cell-cell communication, or clustering of spatial regions can be performed with tools such as Giotto Suite,^23^ VoltRon,^24^ SPIN,^25^ Voyager,^26^ squidpy,^27^ liana,^28^ CellChat,^29^ or commot,^30^ among others.^31^ These provide comprehensive online tutorials that are a good starting point for deeper downstream analysis of Open-ST data.

Marker gene identification is conducted through differential expression analysis, revealing the molecular signatures of different cell types or states. We leverage the spatial information in Open-ST data to identify genes that exhibit structured spatial patterns across the tissue, providing insights into tissue organization and functional domains. These can be visualized in 3D, by exporting the processed data, including cluster assignments and/or gene expression values, to a format compatible with ParaView (e.g., voxel models or point clouds). This allows for the creation of interactive 3D visualizations that integrate tissue histology with transcriptomic information (Figure 10D). These can be replicated with the sample data, tools and Jupyter notebooks in our GitHub repository (https://github.com/rajewsky-lab/openst).

## Limitations

Open-ST is currently limited to the use of fresh-frozen tissue. Use of fixed-frozen or formalin-fixed paraffin-embedded (FFPE) tissue would require protocol adaptations. Fresh-frozen tissue can vary widely in its RNA quality, with time until freezing being a key factor. Since Open-ST is based on poly-dT priming it depends on high RNA quality. Significant RNA degradation will decrease library input and size. Open-ST to date allows the capture of all polyadenylated transcripts; however, it could be adapted for different uses such as the targeted capture of specific sequences.

Open-ST exhibits a strong 3′ bias in gene detection and may underquantify intronic reads, possibly also due to insufficient permeabilization of nuclei during the capture process. This bias should be considered when interpreting gene expression data, particularly for genes with important splice variants or when studying pre-mRNA dynamics. Thus, we discourage applying RNA velocity methods for the analysis of these data.

The spatial coverage of Open-ST is limited by the presence of small gaps between capture area tiles across columns and rows (see Figure 3), which may result in discontinuities in the spatial gene expression map (around 5% of total area). Additionally, the current computational pipeline for cell segmentation is based on nuclear segmentation and radial extension; thus, it may not always accurately represent cellular boundaries, e.g., in neurons. Extensions of Open-ST to include a cell membrane staining could mitigate this limitation.

The resolution discrepancy between the x-y plane (0.6 μm) and z-axis (typically 10 μm) can lead to mixed signals from multiple cells, complicating single-cell analyses. This issue is universal to other ST technologies, and thus is solvable upon future developments in computational tools. Especially, in cell-dense tissue regions, such as lymph nodes, or when the cell diameter is smaller than the tissue section thickness, the resulting single-cell profiles may lack clarity due to signal mixing. More generally, common normalization and dimensionality reduction methods may not fully account for spatial biases, such as lateral diffusion or autocorrelated background signal. These factors can influence clustering and differential expression analyses, potentially leading to spurious cell type identification or inaccurate gene expression results. Users should interpret spatial patterns in light of tissue anatomy and function, validate cluster assignments using known marker genes, and be aware of potential batch effects.

The 3D alignment process in Open-ST does not fully utilize histological information and is limited to rigid transformations. This can be problematic when dealing with tissue samples that have significant folds or are fragmented, potentially leading to misalignment in 3D reconstructions.

Lastly, spacemake as of v.0.7.9 does not include barcode error correction with Hamming distance zero. This limitation may result in the loss of a small fraction of valid spatial barcodes, slightly reducing the overall sensitivity of the method; we estimate around 10% of reads cannot be assigned to spatial locations due to sequencing error. However, implementing barcode error correction with Hamming distance 1 would only recover an additional 5–10% of the data, while significantly increasing computational complexity. Given this modest gain, the current approach represents a balanced trade-off between sensitivity and computational efficiency.

## Troubleshooting

While this section addresses common issues, we maintain an active online community for ongoing discussions and updates. For experimental concerns, please refer to our discussion forum (https://gitter.im/openst/community); for computational matters, consult our GitHub repository (https://github.com/rajewsky-lab/openst). These resources provide the most current solutions and community-driven insights.

### Problem 1

Bubbles form when pipetting into the flow cell for capture area generation (Related to steps 1–12).

### Potential solution

To prevent bubbles from forming in the flow cell lanes, pipette slowly and evenly using a P1000 pipette. Thoroughly remove the previous solution from the lanes before adding new solutions. Do this by repeated pipetting with a P1000 set to 1 mL (both by suctioning out and blowing through air). A vacuum aspirator can also be used.^32^

If bubbles persist, you may consider repeating the incubation for at least 5 h with fresh DraI mix or ExoI, to ensure efficient digestion in all areas. Mark the bubbles with a marker to ensure no bubbles occur at the same place in the second incubation.

Alternatively, you can open the flow cell before Step 3 and perform reactions on the opened flow cell surface, although this would require larger reaction volumes.

### Problem 2

The opened flow cell breaks unevenly into the capture areas (Related to step 14).

### Potential solution

Apply even pressure with the glass cutting tool and score the glass by sliding over it only once. This creates a singular score, which becomes the path of least resistance along which the glass will break. The sharp edge of the cutting wheel should be perpendicular to the glass.

To break the flow cell, place a straight-edged hard surface (like our hand-held breaking aid) parallel to the score on the edge of the flow cell and apply even pressure downwards. The further away from the score you place the breaking aid, the less pressure you need to apply (leverage). Thus, larger capture areas are easier to break, i.e., require less pressure to be applied.

Practicing once on a used (waste) flow cell will give you a feeling on how much pressure to apply and flow cell breaking will soon become a routine procedure.

### Problem 3

Unable to detect the active side, i.e., the side with capture oligos, of your capture area ( Related to step 15).

### Potential solution

The active side can be distinguished from the inactive side by visual inspection. When moving the dry capture area in the light the active side has an iridescent and slightly matt appearance (Figure 1E).

If unsure, the capture area can be inspected under a microscope. The plane with circular alignment marks is the active side.

Make sure to mark the inactive “lower” side of your capture area with an “L.” on the glass area, whilst breaking the flow cell into capture areas and to immediately stick the inactive side of the capture area onto a piece of cut-up plate sealing tape.

### Problem 4

The recommended cycle number is high (>15 cycles) or the cDNA yield is low (Related to step 65 or step 70).

### Potential solution

A low cDNA yield may result from a low input due to a small tissue section, degraded RNA or insufficient permeabilization.

Increase the RNA input by increasing tissue coverage of the capture area or by selecting more cellularized regions. For permeabilization conditions and RNA quality control refer to before you begin section “RNA quality control”, and step-by-step method details section “permeabilization and reverse transcription”. Ensure that self-made buffers (particularly the 2× SSC from the materials and equipment setup section) have the correct pH. Ensure that you remove RNases from your working area and tools.

To exclude insufficient permeabilization or issues with the capture areas as causes, you can add a capture area incubated with a droplet of extracted total RNA (300–500 ng) as a positive control.

If the qPCR cycle number is low (<15), but the cDNA yield is insufficient, you can repeat the protocol from 2nd strand synthesis and amplify with one more cycle (make sure not to pool the second strand from different reactions!).

### Problem 5

The final library contains peaks (Related to step 73).

### Potential solution

If the library shape is not smooth and contains several peaks >300 bp it is possible that the library is of low complexity and underwent over-amplification. If this occurred you can refer to problem 4 or, if your PCR yield was high, repeat the second strand synthesis and amplify the library using fewer cycles.

Any peak <250 bp is to be considered an artifact, since the entire construct length already makes up 226 bp without the cDNA insert. These small artifacts are removed by the gel-based size selection; however, if small peaks persist an additional AmPure XP clean-up at a ratio of 0.6× or 1× beads to product can be performed.

Small artifacts can be primer dimers or randomly primed capture oligo that was not efficiently digested by exonuclease I. Too little input may increase primer dimer formation, thus you may decrease primer concentration or increase input by increasing tissue coverage.

### Problem 6

Automatic alignment of the imaging and transcriptomics modalities fails to work properly (related to Step 95).

### Potential solution

This can be due to challenging tissue morphology.

There are several parameters in the *pairwise_aligner* command that can be fine-tuned: (1) decrease --pseudoimage-size-coarse (or --pseudoimage-size-fine) to create pseudoimages that focus on large-scale features of the tissue structure, instead of small-scale features; (2).

adjust --threshold-counts-coarse and --threshold-counts-fine to filter out noise in low-expression areas; (3) increase --ransac-coarse-max-trials and --ransac-fine-max-trials to allow more attempts at finding a good alignment, and decrease --ransac-coarse-residual-threshold and --ransac-fine-residual-threshold to make the alignment more stringent.

If issues persist, consider using the manual alignment tool for more precise control – manual alignment typically takes less than 1 min per tile.

### Problem 7

Segmentation detects too few cells, especially in areas with high cell density (Related to Step 101).

### Potential solution

Adjust Cellpose parameters, i.e., decrease --cellprob-threshold to detect more potential cells, and increase --flow-threshold for better separation of adjacent cells. Also, set an appropriate --diameter parameter based on the average cell size in your tissue.

For tissues with varying cell sizes, when a population of cells is under-segmented, consider using the two-step segmentation strategy, as used for the segmentation of large adipocytes in the reference Open-ST data.^1^ These segmentation masks can be merged into a single one using openst segment_merge.

### Problem 8

Staining images (H&E) show uneven focus and irregular spatial artifacts (Related to Step 102).

### Potential solution

Run the image_preprocessing script from the openst suite.

>openst image_preprocessing \

> --image-in path/to/image.tif \

> --image-out path/to/output.tif

This applies a machine learning model to improve image quality prior to segmentation. Importantly, this model has been tested on H&E images, only.

### Problem 9

Poor spatial coverage of a sample – all or some tiles are missing from the spacemake output (Related to Step 91).

### Potential solution

Reconfigure the sample in spacemake by adding all tiles again to ensure complete coverage, and reconfigure the spatial_barcode_min_matches threshold to a value lower than currently configured (0.1 by default). This allows inclusion of tiles with less area covered. Re-run the spacemake pipeline with the updated configuration. Only new tiles will be processed – mapping to reference genomes and already quantified tiles will be kept from the previous run if no other parameters are changed.

### Problem 10

Low counts or gene coverage per cell (e.g., <1000 UMI or genes per cell).

### Potential solution

This might indicate insufficient sequencing depth. Check PCR bias values – if below 5, deeper sequencing may improve UMI and gene detection rates. Generate saturation curves running spacemake run downsample to assess if additional sequencing would be beneficial.

## Resource availability

### Lead contact

Further information and requests for resources and reagents should be directed to and will be fulfilled by the lead contact, Nikolaus Rajewsky (rajewsky@mdc-berlin.de).

### Technical contact

Technical questions on executing this protocol should be directed to and will be answered by the technical contact, Marie Schott, Daniel León-Periñán and Elena Splendiani (marie.schott@mdc-berlin.de, daniel.leonperinan@mdc-berlin.de, elena.splendiani@uniroma1.it).

### Materials availability

The designs of the 3D-printed cutting guides and the breaking aid are provided as Items S1, S2, and S3. All other materials used are commercially available.

### Data and code availability

Open-ST RNA-seq and microscopy data used in this study have been downloaded from the publicly available GEO records (accession number GSE251926). All code for spacemake, openst, stimwrap and STIM has been obtained from their original repositories, at https://github.com/rajewsky-lab/spacemake (accessed 20 September 2024), https://github.com/rajewsky-lab/openst (accessed 20 September 2024), https://github.com/rajewsky-lab/stimwrap (accessed 20 September 2024) and https://github.com/preibischlab/STIM (accessed 20 September 2024), and are publicly available as of the date of publication. The Open-ST package v.0.2.3 is archived at https://doi.org/10.5281/zenodo.14197712.

## Acknowledgments

We thank all members of the Rajewsky lab for critical and helpful discussions during the development of Open-ST. Moreover, we thank all users of Open-ST for their feedback, whether online or in person. We thank Alexandra Tschernycheff and Margareta Herzog for supporting the organization of the lab. We thank the Genomics Technology Platform at the MDC/BIH@Charité, particularly Tatiana Borodina and Jeannine Wilde, for performing sequencing runs and providing us with custom flow cells.

Some illustrations in the graphical abstract were created with BioRender.

M.S. was financially supported by the Berlin Institute of Health at Charité (BIH) between December 2020 and February 2022, D.L.-P. was supported by the Helmholtz Einstein International Berlin Research School in Data Science (HEIBRiDS) program of the Helmholtz Association, and E.S. received an Erasmus fellowship. E.S., E.F., and G.M. acknowledge funding from the European Union - The National Recovery Plan, Mission 4 Component 2 Investment 1.4 – Next Generation EU project CN3 – code CN_00000041. G.M. received financial support via a BIH Visiting Professorship “Single Cell Genetics and Epigenetics of Patient Derived Tumor and Brain Organoids” by the Stiftung Charité from 2018 to 2022. N.K. was financially supported by the Deutsche Forschungsgemeinschaft (DFG), grant number KA 5006/1-1. N.R. thanks the Leibniz prize of the Deutsche Forschungsgemeinschaft (DFG) (grant number RA 838/5-1), the DFG Exzellenzcluster 2049 NeuroCure, the Deutsches Zentrum für Herz-Kreislauf-Forschung (DZHK) (grant numbers 81Z0100105 and 81X2100155), and the Chan Zuckerberg Foundation (CZI)/Seed Network.

## Author contributions

M.S., D.L.-P., and E.S. wrote the original draft with input from all authors. M.S. and E.S. performed, wrote, and illustrated the experimental protocol. D.L.-P. performed, wrote, and illustrated the computational protocol, with input from N.K. E.F., G.M., N.K., and N.R. supervised the project. All authors revised the manuscript and approved the final version.

## Declaration of interests

E.F., N.K., G.M., N.R., M.S., and E.S. are listed as inventors of a patent application relating to the work. The patent application was submitted through the Technology Transfer Office of the Max Delbrueck Center, with the MDC being the patent applicant.

## Supplemental information

Document S1. Figure S1 and Item S6

Item S1. Cutting guide 3D-design file for the NovaSeq S4 flow cell, related to steps 18–21 of “Before you begin”

Item S2. Cutting guide 3D-design file for the NovaSeq SP and S1 flow cells, related to steps 18–21 of “Before you begin”

Item S3. 3D-design file of the breaking aid for capture area preparation, related to steps 18–21 of “Before you begin”

Item S4. Open-ST custom sequencing recipe for the NovaSeq 6000 S4, related to step 22 of “Before you begin”

Item S5. Open-ST custom sequencing recipe for the NovaSeq 6000 SP, related to Item S6
