# Supplementary material for: Protocol for high-resolution 3D spatial transcriptomics using Open-ST
Source: STAR Protoc. 2024 Dec 19;6(1):103521. doi: 10.1016/j.xpro.2024.103521 (PMC11731217; doi:10.1016/j.xpro.2024.103521)

Supplementary Item 6: Open-ST capture area generation using the Illumina NovaSeq SP flow cell

To prepare a Open-ST barcoded flow cell, sequence HDMI32-DraI library on an Illumina® NovaSeq 6000 SP flow cell (100 cycles), as described in the Before you Begin section “Barcode sequencing for Open-ST capture area generation”, however, importantly, use the custom sequencing recipe for the SP flow cell (Supplementary Item 5).

Process the sequenced SP flow cell as described in the Step-by-step method details “Preparation of Open-ST capture areas” steps 1-11, using less volume for incubations. Around 75 uL per lane is required per SP flow cell lane (2 lanes).

Open the SP flow cell and denature the second strands as in Step-by-step method details “Preparation of Open-ST capture areas” steps 12 and 13. Unlike the S4 flow cell, only the bottom (thick) surface of the SP flow cell is imaged. Dispose of the top (thin) flow cell surface.

Score and break the SP flow cell into capture areas of your desired size, using the SP cutting guide (Supplementary Item 2) on the bottom flow cell layer. The NovaSeq 6000 SP flow cell consists of 2 lanes. Of each lane only ⅔ of the width is imaged (two of three possible swaths). The unimaged area contains barcoded spots for which we do not have the sequencing information. Consequently, any mRNA captured in this area will not be possible to spatially map. Thus, it is required to physically cut off the unimaged third. To do this, place the SP flow cell barcode face down, oriented as in Supplemental Figure 1 (use the flow cell’s QR code for orientation). Score and break the flow cell as detailed for the S4 in Step-by-step method details “Preparation of Open-ST capture areas” steps 14a. Break off and dispose of the third of the flow cell indicated in Supplemental Figure 1. Store the remaining ⅔ of the lanes for future use at -20°C, following steps 15-17 of Step-by-step method details “Preparation of Open-ST capture areas”.

Note: The dimensions of the NovaSeq 6000 S1 flow cell are identical to the SP flow cell. However, the S1 flow cell as both flow cell surfaces sequenced. Thus, both the top and bottom flow cell layer can be used. Additionally, the SP cutting guide can also be used for the S1 flow cell. However, the location of the unimaged third per lane still has to be confirmed; we assume the orientation is the same as in the SP flow cell.

Supplementary Figure 1: Scheme of NovaSeq 6000 SP flow cell cutting using the 3D-printed cutting tool, related to Supplementary Item 6.

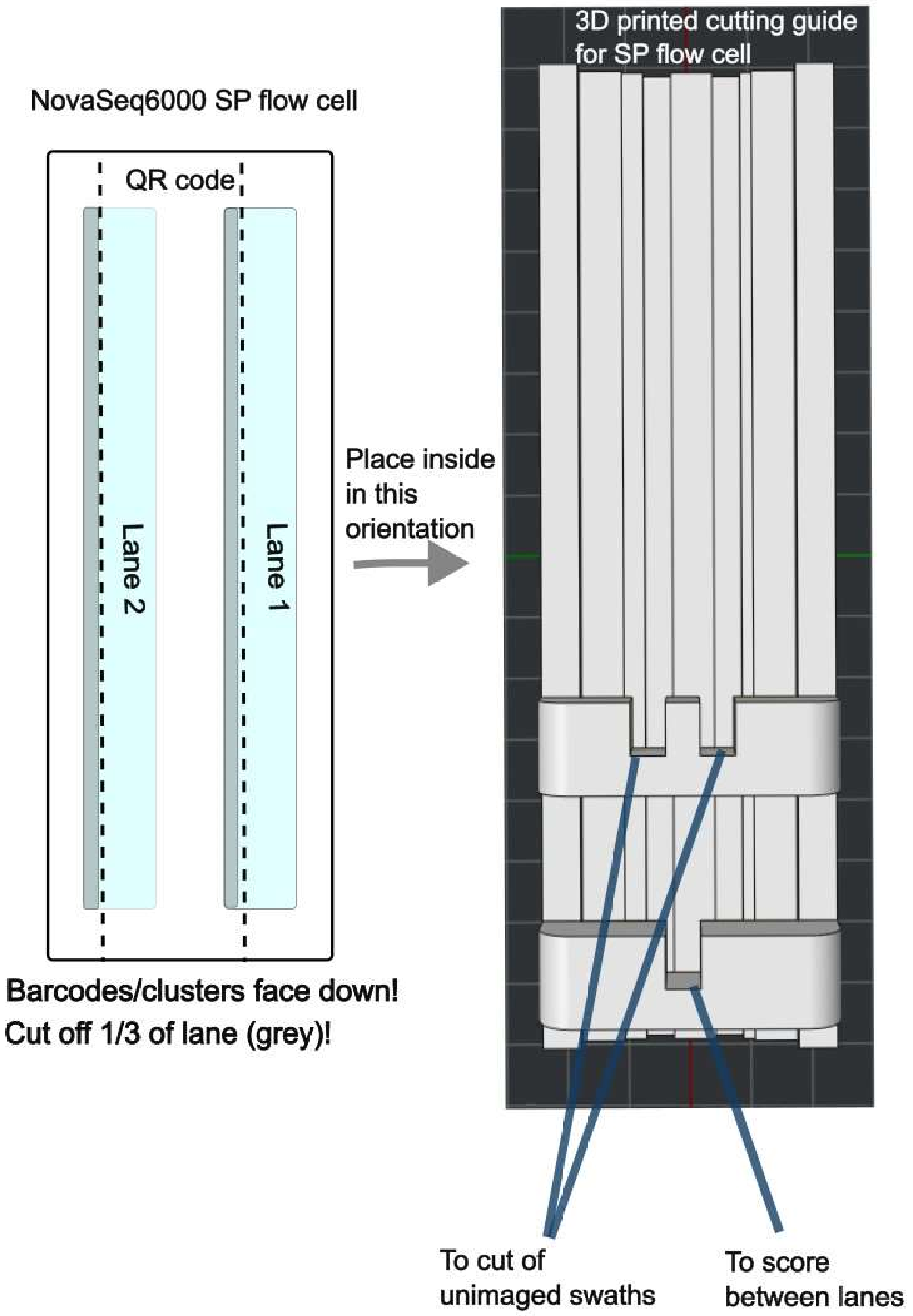

Supplement: Document S1. Figure S1 and Item S6 [file mmc1.pdf]
